# Large-Scale Hydrogen Storage in Deep Saline Aquifers: Multiphase Flow, Geochemical–Microbial Interactions, and Economic Feasibility

**DOI:** 10.3390/ma18225097

**Published:** 2025-11-10

**Authors:** Abdullahi M. Baru, Stella I. Eyitayo, Chinedu J. Okere, Abdurrahman Baru, Marshall C. Watson

**Affiliations:** 1 Bob L. Herd Department of Petroleum Engineering, Texas Tech University, Lubbock, TX 79409, USA; stella.eyitayo@ttu.edu (S.I.E.); marshall.watson@ttu.edu (M.C.W.); 2 Department of Petroleum Engineering, University of Houston, Houston, TX 77204, USA; 3 School of Building Construction, Georgia Institute of Technology, Atlanta, GA 30332, USA; abaru8@gatech.edu

**Keywords:** underground hydrogen storage, deep saline aquifers, hydrogen-brine-rock interactions, levelized cost of storage, cushion gas, geochemical and microbial effects

## Abstract

The development of large-scale, flexible, and safe hydrogen storage is critical for enabling a low-carbon energy system. Deep saline aquifers (DSAs) offer substantial theoretical capacity and broad geographic distribution, making them attractive options for underground hydrogen storage. However, hydrogen storage in DSAs presents complex technical, geochemical, microbial, geomechanical, and economic challenges that must be addressed to ensure efficiency, safety, and recoverability. This study synthesizes current knowledge on hydrogen behavior in DSAs, focusing on multiphase flow dynamics, capillary trapping, fingering phenomena, geochemical reactions, microbial consumption, cushion gas requirements, and operational constraints. Advanced numerical simulations and experimental observations highlight the role of reservoir heterogeneity, relative permeability hysteresis, buoyancy-driven migration, and redox-driven hydrogen loss in shaping storage performance. Economic analysis emphasizes the significant influence of cushion gas volumes and hydrogen recovery efficiency on the levelized cost of storage, while pilot studies reveal strategies for mitigating operational and geochemical risks. The findings underscore the importance of integrated, coupled-process modeling and comprehensive site characterization to optimize hydrogen storage design and operation. This work provides a roadmap for developing scalable, safe, and economically viable hydrogen storage in DSAs, bridging the gap between laboratory research, pilot demonstration, and commercial deployment.

## 1. Introduction

**Figure 1:**
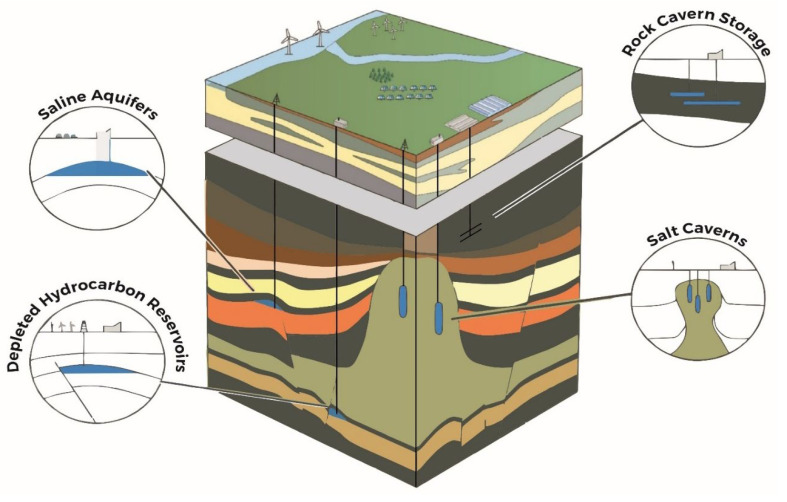
Geological storage solutions.

The transition to a low-carbon energy system requires the development of large-scale, flexible, and geographically distributed energy storage solutions [1,2]. Hydrogen has emerged as a promising energy vector due to its high energy density and compatibility with renewable electricity generation. Among subsurface storage options (Figure 1), deep saline aquifers (DSAs) offer immense theoretical capacities, widespread availability, and potential for seasonal and interannual hydrogen buffering. Unlike mature storage technologies such as salt caverns or depleted gas reservoirs, the exploitation of DSAs for hydrogen storage remains critical, presenting a complex interplay of technical, geochemical, microbial, and economic challenges [3,4].

Hydrogen injection into DSAs is governed by multiphase flow dynamics, where low viscosity and high mobility relative to brine can lead to viscous and capillary fingering, uneven displacement, and residual trapping [5,6,7]. Simultaneously, geochemical reactions between hydrogen, formation minerals, and brine can consume hydrogen, produce secondary gases such as hydrogen sulfide, and alter reservoir properties. Indigenous microbial populations further exacerbate hydrogen loss through methanogenesis and sulfate reduction, potentially affecting gas purity and reservoir integrity [8]. Additionally, operational requirements, including cushion gas management, cyclic injection, and withdrawal strategies, impact hydrogen recoverability and economics. Geomechanical responses, caprock integrity, and induced seismicity risks must also be addressed to ensure safe, long-term storage.

While pilot studies and laboratory investigations have begun to elucidate these processes, integrated assessments combining multiphase flow, geochemical–microbial interactions, geomechanics, and economic feasibility remain limited. Understanding how these factors interact under realistic reservoir conditions is critical for designing robust, scalable hydrogen storage systems.

The scientific and technical foundation for hydrogen storage in deep saline aquifers has been established by the geological storage of carbon dioxide (CO_2_). We now have a better understanding of caprock integrity and subsurface fluid dynamics thanks to emerging CO_2_ storage technologies like risk-based management, advanced modeling, and AI-assisted monitoring [9]. These developments provide insightful information that can be applied to large-scale hydrogen storage, where containment, migration, and monitoring issues are still prevalent.

The primary objective of this study is to provide a comprehensive analysis of hydrogen storage in deep saline aquifers by integrating multiphase flow behavior, geochemical and microbial interactions, geomechanical stability, operational constraints, and economic considerations. This work aims to synthesize current knowledge, identify key technical and operational challenges, and propose strategies for efficient and safe large-scale hydrogen storage in DSAs.

Given the increasing demand for dependable large-scale hydrogen storage, this review aims to address the research question: what effects do geochemical–microbial reactions, coupled multiphase flow dynamics, and financial limitations have on the viability, safety, and efficiency of large-scale hydrogen storage in deep saline aquifers? To answer this question, these review-specific objectives were applied:

- To compile the most recent knowledge on capillary, residual, and structural trapping processes as well as hydrogen transport and trapping mechanisms in deep saline aquifers.
- To assess how microbial activity and geochemical reactions affect the long-term integrity of reservoirs, storage capacity, and hydrogen purity.
- To examine the effects of operational elements on storage performance and recovery efficiency, including reservoir heterogeneity, pressure cycling, and cushion gas composition.
- To link reservoir behavior with levelized storage costs and system scalability in order to evaluate the techno-economic viability of large-scale subterranean hydrogen storage.
- To determine the most important areas for future study and the steps that must be taken in order to advance safe, effective, and financially feasible hydrogen storage in deep saline aquifers.

Consequently, the goal of this review is to present a comprehensive understanding of the interrelated hydro-biogeochemical and techno-economic processes that control large-scale underground hydrogen storage (UHS) in deep saline aquifers. It summarizes the most recent findings on capillary trapping, multiphase flow dynamics, and microbial–geochemical interactions that affect hydrogen behavior in subsurface settings. In order to determine the crucial elements that govern the effectiveness, scalability, and long-term stability of hydrogen storage systems, it also assesses operational, safety, and financial factors, such as cushion gas design and levelized cost of storage.

## 2. Current Status of Hydrogen Storage

The global hydrogen economy increasingly depends on reliable, efficient storage solutions that span from transport fueling to grid-scale seasonal buffering. Broadly, hydrogen storage technologies can be grouped into compressed gas, liquid hydrogen, solid-state materials, and geological reservoirs. Each method presents unique trade-offs in energy density, system complexity, cost, and longevity.

### 2.1. Storage Methods Overview

Compressed gas storage in high-pressure tanks remains the most mature technology for onboard and short-term applications. Leading fuel cell vehicles routinely utilize composite Type IV tanks rated at 700 bar, achieving gravimetric energy densities of approximately 1.4 kWh/kg and volumetric densities near 0.8 kWh/L [10]. Despite wide uptake, compression energy penalties (around 5–10%) and system-level costs (estimated at ~USD 400–700 per kg H_2_) limit broader scale deployment [11].

Liquid hydrogen offers a markedly higher volumetric density (~8.5 MJ/L), making it suitable for bulk transport and aerospace applications. However, liquefaction consumes significant energy (around 13–14 kWh/kg), and cryogenic boil-off losses typically range from 1 to 5% per day unless active refrigeration or advanced insulation solutions are employed.

Solid-state storage covers both physisorption (e.g., activated carbon, MOFs) and chemisorption (e.g., metal hydrides, LOHCs). Physisorption materials deliver moderate densities within cryogenic or high-pressure regimes, whereas metal hydrides such as MgH_2_ or LaNi_5_ offer reversible storage capacities of 5–7 wt %, though often at elevated temperatures (100–300 °C) and with kinetic limitations and high material costs (USD 2000–5000/kg H_2_) [12].

#### 2.1.1. Compressed Gas

Hydrogen stored under high pressure in composite tanks is widely deployed in both stationary applications and transportation. Commercial Type IV tanks operating at 700 bar provide gravimetric densities up to ~1.4 kWh/kg and volumetric storage capacities near 0.8 kWh/Lss [10]. While energy consumption during compression reaches 5–10% of input energy, system maturity and infrastructure support continue to make this method the default choice for light-duty mobility and refueling applications. Ongoing R&D focuses on lightweight liners, improved sealing technologies, and reduced tank costs to approach DOE targets.

#### 2.1.2. Liquid Hydrogen

Liquefied hydrogen achieves the highest volumetric density among physical states (~70.8 kg/m^3^ or ~8.5 MJ/L). Its use is predominant in aerospace and long-haul transport sectors where cryogenic challenges are acceptable. However, liquefaction incurs a high energy penalty (~13–14 kWh/kg) and daily boil-off can range from 1 to 5%, unless advanced thermal management (e.g., self-pressurizing systems like Toyota’s) is integrated [13]. The requirement for cryogenic infrastructure and insulation limits its practicality in mobile or decentralized systems.

#### 2.1.3. Solid-State Storage

Solid-state hydrogen storage leverages materials-based mechanisms to reversibly store hydrogen. Metal hydrides (e.g., MgH_2_, LaNi_5_, and emerging high-entropy alloys) chemisorb hydrogen with capacities up to ~7 wt %, but typically require elevated temperatures and suffer from slow kinetics and large weight penalties. Physisorption using MOFs or high-surface-area carbons enables hydrogen uptake at low temperatures (~100 K), but storage capacity falls sharply at ambient temperature and tight cycling constraints limit their application [10]. Liquid organic hydrogen carriers (LOHCs) such as methanol or aromatic systems offer large-scale storage potential with round-trip efficiencies of 60–90%, yet require catalytic infrastructure and face challenges around hydrogen liberation rates and system integration.

According to Le et al. [14], one of the biggest obstacles to developing a sustainable hydrogen economy is still hydrogen storage. Their analysis of physical hydrogen storage techniques, such as advanced sorbent materials, compressed gas, and cryogenic storage, showed that the effectiveness, scalability, and safety of hydrogen energy systems are all influenced by the storage medium selection. The performance of hydrogen uptake and release is improved by materials-based storage systems, such as metal–organic frameworks (MOFs), zeolites, and carbon nanostructures, which have superior surface area and adjustable pore structures. In porous geological formations such as deep saline aquifers, where pore geometry, connectivity, and wettability regulate hydrogen entrapment and migration, these engineered materials offer helpful analogs. Thus, the design and predictive modeling of subsurface hydrogen storage behavior in natural formations can benefit from an understanding of the interactions between hydrogen and the internal pore network in synthetic materials.

### 2.2. Geological Storage

Geological storage represents an important pathway for achieving large-scale, long-duration hydrogen storage. Unlike engineered systems such as compressed tanks or cryogenic vessels, geological formations offer volumetric capacities orders of magnitude greater, suitable for seasonal balancing, backup power, and industrial buffering. The subsurface formations considered for hydrogen storage include deep saline aquifers, depleted oil and gas reservoirs, salt caverns, and, to a lesser extent, abandoned mines. Each of these options offers distinct advantages and presents specific challenges associated with containment integrity, geochemical compatibility, injectivity, and retrieval efficiency.

#### 2.2.1. Saline Aquifers

Deep saline aquifers represent the most abundant and geographically widespread option for underground hydrogen storage. These formations consist of porous rock saturated with non-potable brine and are typically found at depths exceeding 800 m, where pressure and temperature conditions favor hydrogen containment in the supercritical or gaseous state [15]. Their principal advantage lies in the immense volumetric storage potential and strategic distribution near industrial hubs and renewable energy assets.

However, several challenges complicate the storage of hydrogen in saline aquifers. The main issue is the reactive nature of hydrogen with formation fluids and minerals, which may lead to microbial activity (e.g., sulfate-reducing bacteria), pH shifts, and mineral dissolution or precipitation. These geochemical and biogeochemical reactions can reduce porosity, impair injectivity, and affect hydrogen purity upon withdrawal. Furthermore, the low viscosity and high diffusivity of hydrogen increase the risk of caprock leakage unless sealing formations are thoroughly characterized and validated.

#### 2.2.2. Depleted Oil and Gas Reservoirs

Depleted oil and gas reservoirs offer an attractive option for hydrogen storage due to their well-documented geological characteristics, existing infrastructure (e.g., wells, pipelines), and proven containment over geological time scales. These formations usually consist of porous sandstones or carbonates that have previously held hydrocarbons under pressure, implying favorable porosity, permeability, and structural closure.

The re-use of depleted reservoirs for hydrogen storage can significantly lower capital investment and expedite permitting, especially where legacy data are available. Additionally, residual hydrocarbons and cushion gases in these formations may reduce microbial activity compared to aquifers. However, several challenges exist. First, the compatibility of hydrogen with residual hydrocarbons and formation brine must be evaluated, particularly regarding phase behavior, wettability alteration, and gas solubility. Second, potential leakage pathways such as old wellbores, faults, or fractures must be remediated or monitored to ensure containment integrity.

#### 2.2.3. Salt Caverns

Salt caverns are currently the most technologically mature option for underground hydrogen storage and are already employed for large-scale hydrogen and natural gas storage in countries such as the United States, Germany, and the United Kingdom. These caverns are typically created by solution mining of bedded or domal salt formations, resulting in stable voids with high containment integrity due to the self-healing and low-permeability nature of salt.

Hydrogen stored in salt caverns benefits from several key advantages: high injection and withdrawal rates, minimal cushion gas requirements, and relatively low contamination risk. Moreover, salt caverns can withstand multiple operational cycles annually, making them ideal for balancing intermittent renewable energy sources such as wind and solar [16]. Gravimetric energy densities in such systems can reach up to 6 MJ/kg, with cavern volumes ranging from 100,000 to over 1,000,000 cubic meters [16].

The major limitations of salt caverns include their high capital cost for construction, geographic constraints (they occur only in specific geological settings), and limited total storage volume per cavern. Nonetheless, their operational reliability and proven safety record make them the preferred choice for commercial-scale hydrogen storage today.

#### 2.2.4. Abandoned Mines

Abandoned or disused mines have also been considered as potential hydrogen storage sites due to their existing underground void space and accessibility. These include coal mines, metal ore shafts, and other extractive works that may extend to depths suitable for pressure containment. The use of such facilities could reduce surface footprint and leverage existing access infrastructure.

However, the feasibility of hydrogen storage in abandoned mines remains largely theoretical and faces significant technical and environmental barriers. The fractured and heterogeneous nature of mine shafts often leads to high leakage potential, especially if sealing and grouting are inadequate [17]. Additionally, the risk of undesirable geochemical reactions, residual contaminants, and microbial activity is elevated in the formation [18].

While small-scale, localized energy storage applications might be considered, the long-term stability and safety of hydrogen storage in abandoned mines are uncertain. Detailed site characterization, sealing strategies, and regulatory oversight would be essential before it can be considered a viable alternative to engineered or natural porous formations.

### 2.3. Comparative Analysis of Hydrogen Storage Options

The selection of a hydrogen storage method is dictated by a complex interplay of technical, economic, and spatial considerations. As the global hydrogen economy matures, the need for flexible, scalable, and safe storage becomes increasingly urgent, not only for balancing production and demand but also for supporting energy system resilience and decarbonization. While physical and chemical storage methods such as compressed gas, liquid hydrogen, and solid-state materials offer portability and high-purity retrieval, their volumetric limitations and cost barriers constrain their role to short-term and small-scale applications. In contrast, geological storage in subsurface formations provides a pathway to storing large amounts of energy for long durations, but introduces challenges related to site-specific variability, gas-rock interactions, and retrieval predictability.

Compressed hydrogen gas is the most technologically straightforward approach and benefits from commercial maturity, modularity, and compatibility with existing infrastructure. However, it is limited by low volumetric energy density and high compression energy costs. Liquid hydrogen, while offering greater energy density, requires extreme cryogenic conditions, resulting in boil-off losses, high insulation demands, and significant lifecycle energy penalties. Solid-state storage materials, such as metal hydrides or porous sorbents, remain promising for onboard and stationary applications due to their high volumetric densities and safety profiles, but most materials have yet to overcome barriers related to slow kinetics, limited reversibility, and high weight fractions.

Geological storage options fundamentally differ in scale and dynamics. Salt caverns currently represent the most commercially viable geological option. Their fast cycling rates and low contamination risks make them ideal for high-frequency storage. However, their geographic distribution is limited, and their construction costs are substantial. Depleted oil and gas reservoirs, by contrast, are widespread and benefit from legacy infrastructure and detailed geological characterization, though they raise concerns about gas purity and leakage through legacy wells. Saline aquifers offer the greatest theoretical storage capacity and geographic flexibility, but remain largely untested at commercial scale for hydrogen, with key uncertainties surrounding geochemical reactivity, microbial activity, and plume behavior. Abandoned mines are the least understood option, with high heterogeneity and containment-related challenges.

Table 1 synthesizes the key attributes of the major hydrogen storage pathways. It highlights the comparative advantages of geological storage for large-scale, seasonal balancing applications, while illustrating the utility of engineered storage options in mobile, distributed, or urban contexts. No single method is universally optimal; rather, a hybrid approach tailored to specific use-cases may represent the most pragmatic strategy.

The comparative analysis shows that the suitability of a hydrogen storage technology must be assessed within specific energy system demands. Applications requiring rapid deployment, compactness, and high purity, such as vehicular fuel cells or backup power, favor compressed or liquid storage. Conversely, grid-scale energy arbitrage, renewable integration, and strategic energy security objectives demand the volumetric and temporal flexibility afforded by geological formations. Accelerated field demonstration, integrated techno-economic modeling, and standardized regulatory frameworks will be critical to enable hybridized storage systems and maximize the utility of each method in the broader hydrogen value chain.

## 3. Subsurface Mechanisms of Hydrogen Storage in Saline Aquifers

The mechanism of hydrogen injected into deep saline aquifers is governed by a complex interplay of physical transport, trapping, and biogeochemical processes that control storage efficiency, injectivity, and long-term containment. Upon injection, hydrogen disperses through porous media via advection and diffusion, influenced by rock heterogeneity, multiphase flow dynamics, and interactions with formation fluids. Reactive transport processes such as mineral dissolution, ion exchange, and microbial metabolism further alter hydrogen mobility and influence porosity and permeability over time. Cushion gases are often employed to mitigate hydrogen loss and maintain pressure, while adsorption–desorption dynamics at the pore scale introduce further complexity. Trapping mechanisms like structural, residual/capillary, and adsorption play critical roles in retaining hydrogen within the formation, with residual trapping being critical during post-injection migration. Additionally, microbial activity in the subsurface can consume or transform hydrogen through methanogenesis, sulfate reduction, or acetogenesis, posing both risks and opportunities for geo-energy integration. Understanding these coupled mechanisms is essential for designing safe, efficient, and scalable hydrogen storage strategies within saline aquifers.

### 3.1. Subsurface Transport and Storage Mechanisms

#### 3.1.1. Reactive-Transport Mechanisms

Reactive transport in hydrogen storage systems involves complex physicochemical processes whereby injected hydrogen traverses, interacts with, and alters the brine–rock matrix. At the pore scale, hydrogen injection into brine-saturated sandstones is characterized by non-wetting behavior, as evidenced by in situ X-ray CT studies that isolate the formation of capillary and viscous fingering fronts [25]. These pore-level mechanisms critically influence recovery efficiencies and define the dynamic retention of hydrogen as residual saturation, thereby determining storage security and withdrawal performance.

Geochemical interactions, though limited in magnitude, are non-negligible over storage lifetimes. Laboratory and modeling studies show carbonate and Fe(III)-bearing minerals near caprock, or pore structures, respond to hydrogen exposure. Specifically, pyrite reduction and calcite dissolution have been detected under reservoir conditions, with modeling suggesting pore-space alterations affecting permeability and caprock integrity [26]. Abiotic mineral alteration appears modest (<5%), yet the formation of hydrogen sulfide requires attention due to corrosion potential, especially in the presence of sulfate-reducing bacteria [27].

Microbial catalysis compounds these concerns. Sulfate-reducing, methanogenic, and iron-reducing microbes can consume up to 40% of injected hydrogen within months, transforming it into methane or hydrogen sulfide and thereby impacting recovery and safety. These reactions simultaneously modify redox states, precipitate secondary minerals, and may clog pore throats. Biogeochemical reactive–transport simulations coupling PHREEQC-modeled dissolution/precipitation and microbial kinetics have become indispensable tools for forecasting storage quality and caprock resilience on a long-term [28].

Lastly, hydrogen dissolution into formation water, though limited in magnitude (≈1–3% per year), further complicates transport behavior, altering both chemical equilibrium and buoyant flow properties. Experimentally derived diffusion coefficients suggest that salinity, pressure, and temperature influence dissolved hydrogen transport and thus warrant inclusion in operational predictive models [29].

#### 3.1.2. Geochemical Reactions

Key trapping mechanisms that guarantee long-term containment have been identified by studies on CO_2_ geological storage, including structural, residual, solubility, and mineralization [9]. Assessments of hydrogen storage are also influenced by these processes, especially when it comes to caprock integrity and geochemical interactions. Assessing hydrogen behavior in deep saline aquifers directly benefits from knowledge of mineral reactivity, porosity evolution, and sealing efficiency gained from CO_2_ storage studies.

Geochemical reactions during UHS in deep saline aquifers occur at the intersection of fluid–rock interactions, redox chemistry, and mineral dynamics. While hydrogen is generally considered minimally reactive with reservoir rocks, field and laboratory studies have shown reactions that influence long-term storage stability and well integrity.

One of the most significant abiotic reactions involves the dissolution and reductive dissolution of carbonates and sulfates. As noted by a previous study, hydrogen–brine contact in calcite-rich sandstones can result in up to 9.5% hydrogen loss over 30 years, driven by pH changes and carbonate dissolution, particularly where the molar ratio of CO_2_ to H_2_ is significant [30]. Similarly, simulations and experiments showed that carbonate (e.g., calcite, dolomite) and sulfate minerals (gypsum, anhydrite, barite) undergo limited dissolution within saline porous media under pressure and temperature conditions typical of UHS, with uneven porosity increases of up to 5% after decades [31]. Hence, reservoirs devoid of reactive carbonates and sulfates exhibit greater chemical resilience and are thus more desirable for hydrogen storage.

Reductive dissolution of Fe(III) minerals, such as hematite and goethite, has also been observed in H_2_-laden brines, but typically requires elevated temperatures or microbial catalysts [31]. Likewise, pyrite shows reductive sensitivity toward hydrogen, converting to pyrrhotite under certain conditions, although this again appears to proceed slowly under typical reservoir conditions [32]. While direct chemical reactivity tends to be minor, these reactions can influence the mechanical properties of caprock and matrix over extended periods, introducing potential risks to storage containment [33].

Microbial activity exerts perhaps an even more significant impact. The saline aquifer supports diverse hydrogenotrophic microbes, especially sulfate-reducing bacteria, methanogens, and iron-reducing organisms that can convert injected hydrogen into methane or hydrogen sulfide, reducing recoverable hydrogen by up to 50% in some studies [34]. The conversion reaction can be represented by Equations (2) and (3).

These biotic pathways not only pose a significant loss of hydrogen but also generate corrosive byproducts (hydrogen sulfide, organic acids) and can induce pore clogging through biomass or precipitates, impacting injectivity and retrieval efficiency [8,35]. Laboratory-scale reactive transport modeling, often implemented via PHREEQC, demonstrates that microbial interactions can accelerate carbonate dissolution and modify porosity-permeability relationships over time [8].

Overall geochemical and microbial reaction extent in aquifer storage scenarios is usually quantified through coupled reactive transport models over 10–30 years, predicting modest rock alteration but potentially high hydrogen losses unless microbial growth is suppressed [8]. Field data confirm this complexity: at the Lobodice town-gas site, nearly 50% of hydrogen was microbially converted to methane within seven months.

#### 3.1.3. Role of Cushion Gases

Cushion gas, the immobile gas volume retained permanently within a subsurface storage formation, plays a critical role in enabling stable hydrogen operations in deep saline aquifers. While hydrogen itself could serve, alternative gases such as methane (CH_4_), nitrogen (N_2_), or carbon dioxide (CO_2_) are more commonly employed due to their favorable thermodynamic properties compared to hydrogen [36].

In porous aquifers, cushion gas ensures reservoir pressure is maintained above the brine entry pressure, preventing water inflow and guaranteeing efficient injection and withdrawal rates. Numerical simulations incorporating compositional equation-of-state models reveal that saline aquifers may require cushion gas volumes of 45–80% of total capacity, significantly more than depleted gas fields (≈50%) or salt caverns (≈20–30%) [37]. This large cushion gas requirement imposes a capital burden and influences operational economics, as cushion gas is non-recoverable.

The type of cushion gas affects both hydrogen recovery efficiency and purity. Simulations comparing N_2_, CH_4_, and CO_2_ under cyclic hydrogen injection show that CH_4_ yields the highest recovery (~77%) and limited hydrogen plume spreading, whereas CO_2_, due to its higher solubility in brine, increases water co-production and reduces recovery (~74%) [37,38]. However, CO_2_ improves purity by suppressing hydrogen diffusion into brine. N_2_ emerges as a balanced choice, offering strong pressure support and moderate recovery (≈74–75%) [38].

Mechanistically, heavier cushion gases help stabilize the displacement front, suppress upward buoyant migration of hydrogen, and maintain a narrower plume, enhancing channeling control [36]. Core-scale experiments confirm that mixing hydrogen with 50% CH_4_ increases relative gas permeability by ~70% and halves the cushion gas volume required to pressurize the core to 1000 psi [39].

Retention and recoverability dynamics evolve with cycling: simulation studies show that recovery improves over successive injection–withdrawal cycles as cushion gas becomes more homogenized and brine displacement stabilizes [40]. However, operational design, such as placement and count of wells can significantly influence efficiency [41].

**Figure 2:**
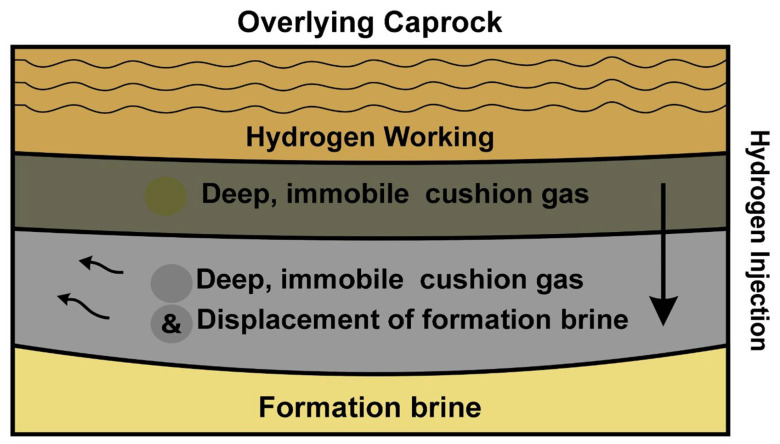
Cushion gas layering under caprock in saline aquifer.

Figure 2 illustrates cushion gas layering under caprock in a saline aquifer: a lower cushion gas zone supports pressure, while overlying hydrogen forms a mobile working plume, displacing brine radially.

Economically, the presence and volume of cushion gas represent a significant non-recoverable investment. Research indicates that hydrogen-based cushion is the most expensive option due to lower density and higher pre-injection volumes required [41]. Using CH_4_ or N_2_ can reduce upfront costs and improve recovery but comes with trade-offs in mixture purity and potential CO_2_ emissions. Optimal cushion gas selection, therefore, represents a multi-objective decision balancing recovery efficiency, storage cost, withdrawal purity, and operational risk.

Therefore, cushion gas function is critical for UHS in saline aquifers. While it reduces working storage capacity, its efficacy in supporting pressure maintenance, enhancing recovery, and enabling controlled hydrogen plume behavior is fundamental. Future research must target strategies to minimize cushion volume via advanced well design and zone isolation, and innovative use of moderate-density gas blends. Such advances will be important in advancing the commercial viability of saline aquifer hydrogen storage.

#### 3.1.4. Adsorption and Desorption of Hydrogen

In addition to hydrodynamic and geochemical interactions, hydrogen storage in deep saline aquifers is influenced by adsorption (a process wherein hydrogen molecules adhere to mineral surfaces even under reservoir conditions). While commonly associated with engineered porous materials like carbon or zeolites, adsorption phenomena have also been observed in natural geological formations comprising organic-rich shales, clay-rich sandstones, and coals [42]. Laboratory studies demonstrate that hydrogen uptake in reservoir rocks is influenced by pressure, temperature, and mineralogy, resulting in significant impacts on storage performance.

In-depth X-ray computed tomography and sorption experimentation on water-wet sandstone reveal significant adsorption isotherm hysteresis: at 298 K, hydrogen uptake ranges from ≈0.2 to 0.8 cm^3^ g^−1^ up to 9 MPa, decreasing with elevated temperatures [36]. This uptake varies greatly based on wettability and pore structure; water-wet rocks exhibit the highest adsorption. It is well-established that adsorption capacity scales with specific surface area and inversely with pore diameter: nanopores (<2 nm) can hold dense hydrogen layers due to overlapping potential from opposing walls.

Coal seams offer a contrasting case, where higher organic content correlates with increased hydrogen adsorption. For instance, sub-bituminous coals demonstrated up to ~0.6 mol H_2_ per kg at 14 MPa, with minimal sensitivity to temperature [43]. Adsorption in clay-rich shales is similarly influenced by organic content and montmorillonite presence, achieving uptake 2–12 times greater than mineral-only matrices under 25 °C and 18 MPa [44]. The isosteric heat of adsorption is modest (~4–14 kJ/mol), consistent with low-energy physisorption processes [45]. Notably, adsorption decreases as hydrogen loading increases, indicating a heterogeneous surface wherein stronger binding occurs at low coverage [45].

However, the practical impact of adsorption on hydrogen storage capacity is minimal. The total adsorbed volume often represents only a few percent of the injected hydrogen during low-pressure withdrawal conditions, where desorption hysteresis can delay output. Recovery losses due to adsorption may range between 2 and 8%, particularly in formations with high specific surface areas [46].

**Figure 3:**
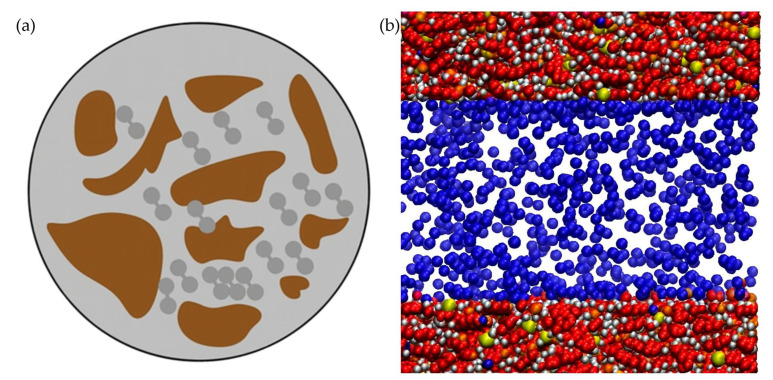
Schematic representation of hydrogen adsorption across different rock types and pore scales (**a**) hydrogen adsorption in clay [coalbed methane] (**b**) hydrogen adsorption in kerogen (modified from [42]).

Figure 3 visualizes hydrogen adsorption across different rock types and pore scales, illustrating enhanced adsorption in nanopores (<2 nm) and organic-rich matrices, with desorption plateauing at lower pressures.

According to Palade et al. [47], a key factor in regulating the behavior of hydrogen desorption and reabsorption is the dispersion quality of nanoscale catalysts. Their research demonstrated that ferrite nanoparticles (NiFeO_4_ and CoFeO_4_) that were uniformly distributed within graphene matrices under inert argon conditions performed better catalytically than those that were synthesized under reducing H_2_/Ar flow. Outperforming all other tested configurations, the enhanced dispersion led to better hydrogen mobility, a lower desorption temperature (~349 °C), and a reversible hydrogen capacity of ~6.14 weight percent. On the other hand, metal clusters and decreased reaction uniformity were the results of reducing atmospheres. This shows how a homogeneous catalytic interface improves gas diffusion and reaction kinetics. This is conceptually similar to how microbial or mineral heterogeneity can change hydrogen migration and consumption rates locally in deep saline aquifers due to uneven spatial distributions of reactive surfaces.

Overall, although adsorption plays a secondary role relative to buoyant displacement in deep aquifers, its influence becomes more significant in organic-rich reservoirs or low-permeability systems. Adsorption contributes to residual trapping, affects hysteresis, and regulates recovery under varying pressure operations. Consequently, practical aquifer hydrogen-storage models must incorporate adsorption–desorption cycles, particularly for scenarios featuring ramped withdrawal or cold-pressure operations.

### 3.2. Trapping Mechanisms of Hydrogen in Geo-Storage Media

A three-dimensional phospho-doped graphene (3D Pd_3_P_0.95_/P-rGO) structure modified by palladium phosphide was created by Chen et al. [48] and greatly improved mass transfer and hydrogen adsorption. In order to improve hydrogen diffusion and storage kinetics, the 3D porous framework was created to reduce graphene sheet aggregation while boosting gas–solid interfacial contact. The optimized composite, which was created using calcination and hydrothermal techniques, had a rapid adsorption equilibrium in two hours and a hydrogen storage capacity of 3.79 weight percent at 298 K and 4 MPa. The strong metal–phosphorus interface and hierarchical pore network, which promoted hydrogen dissociation and spillover, were credited with this improvement. The multiscale pore connectivity and diffusion processes in deep saline aquifers, where interfacial characteristics and reservoir heterogeneity control hydrogen migration, trapping effectiveness, and reversibility during storage operations, are conceptually comparable to these mechanisms.

#### 3.2.1. Structural and Stratigraphic Trapping

Structural and stratigraphic trapping form the primary containment mechanisms for hydrogen stored in deep saline aquifers. When hydrogen is injected into a permeable saline formation, its low density relative to brine drives buoyant migration upward through pore space, pooling beneath an impermeable caprock. In saline aquifers with structural closure such as anticlines, fault-bounded domes, or pinch-outs, the injected gas is confined by overlying layers of low permeability (e.g., mudstone, shale, evaporites), effectively preventing vertical escape.

In the case of salt caverns or structurally closed aquifers, pressure support provided by cushion gas helps maintain reservoir integrity, but in saline aquifers, the presence of a caprock alone suffices to temporarily trap hydrogen. Numerical simulations demonstrate that structural trap efficiency depends strongly on trap geometry, seal strength, and plume sizing relative to trap closure dimensions. For example, a large anticline with a continuous capstone can retain 95–99% of the injected hydrogen for decades. Conversely, small-scale stratigraphic pinch-outs or partially closed faults limit hydrogen volumes and may accelerate lateral seepage, reducing containment unless active pressure management is employed.

Structural and stratigraphic traps collectively ensure both bulk storage and long-term containment of hydrogen. Structural geometry provides a secure initial trap, while capillary forces grant retention at the pore scale. As advances in 3D seismic imaging, capillary pressure testing, and reservoir simulation converge, tailored trap design and strategic well placement are becoming achievable, paving the way for efficient, secure hydrogen storage at the field scale.

#### 3.2.2. Residual/Capillary Trapping

**Figure 4:**
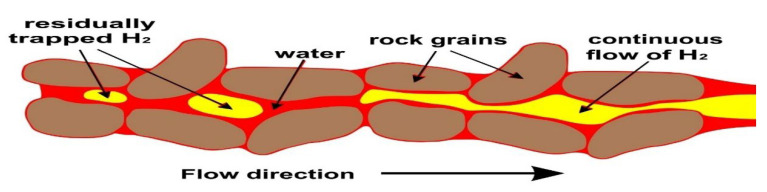
Residual trapping mechanisms in a saline aquifer hydrogen storage system.

Residual or capillary trapping is the process by which hydrogen becomes immobilized in the pore spaces of a reservoir rock due to capillary forces that prevent it from migrating even after pressure support ceases (Figure 4). This mechanism plays a vital role in ensuring storage security and contributes significantly to the overall retention of injected hydrogen in deep saline aquifers.

At the microscale, capillary trapping is governed by the interplay between pore throat geometry and wetting properties (Figure 4). In water-wet sandstone, critical capillary pressure (Pc) thresholds of a few to tens of millibars are sufficient to pin gas bubbles within pore throats, forming disconnected ganglia of hydrogen that remain trapped as pressure declines. This immobilization effect is robust: core-flooding experiments demonstrate that once hydrogen is displaced by brine during cessation of injection, approximately 10–40% of the injected hydrogen volume remains trapped as residual saturation. Notably, this retention increases with smaller pore throat sizes and greater pore surface area. In nanoporous shales and organically enriched sandstones, such can reach up to 60%, especially when hydrogen invades through capillary fingering under low capillary number conditions.

On a field scale, residual trapping stabilizes the hydrogen plume beneath the caprock, significantly increasing containment reliability. Working-gas recovery factors, defined as the fraction of recoverable hydrogen after accounting for residual saturation, can be estimated by:

(1) $$Recovery=\;\frac{S_{r}}{S_{inj}}\;$$

where *S_r_* is the residual saturation achieved during injection, and *S_inj_* is the hydrogen saturation achieved during injection.

The effectiveness of capillary trapping depends heavily on hysteresis behavior: relative permeability and capillary pressure during imbibition differ from those during drainage. Neglecting this hysteresis leads to overestimation of recoverable volumes and underestimation of trapped hydrogen. Hence, advanced models increasingly employ hysteresis-informed curves derived from p–c and k measurements under cyclical wetting conditions.

Empirical data confirm that such residual trapping enhances both containment security and chemical isolation, as entrapped hydrogen is less likely to migrate or interact with overlying strata. However, it places a limit on recoverable working volumes, compelling operators to strike a balance between storage capacity and retrievability.

#### 3.2.3. Adsorption Trapping

Adsorption trapping (where hydrogen molecules adhere to mineral surfaces) is an important mechanism for hydrogen retention in subsurface environments. Though influenced by mechanisms like structural and capillary trapping, adsorption becomes significant in formations characterized by high surface area, such as clay-rich shales, organic-rich sandstones, and zones of authigenic mineral crusting.

Adsorptive capacity is governed by rock mineralogy, pressure, temperature, and pore structure. In clay-rich formations, hydrogen sorption isotherms at 298 K show uptake values between 0.2 and 1 cm^3^ (STP)/g up to 10 MPa. Organic-rich shales and coals demonstrate even greater capacity: lab experiments up to 14 MPa report sorption uptakes of 0.5–0.8 mol/kg in macerals and kerogen-rich samples. These datasets produce typical adsorption energies of 5–15 kJ/mol, confirming physical physisorption rather than chemical bonding. Accordingly, adsorption capacity decreases with temperature but increases with pressure and organic-matter content.

Quantification of adsorptive retention enables assessment of retention fractions. Under reservoir conditions (5–10 MPa, 40–60 °C), adsorbed hydrogen may equate to 2–7% of injected gas in high-organic systems but remains below 1% in clean quartz sandstones. Importantly, adsorption and desorption processes display hysteresis: upon depressurization, desorption is slower, leading to residual adsorbed volumes that reduce working recovery—an effect exacerbated in tight or clay-rich horizons that foster hydrogen accumulation.

Though adsorption trapping comprises only a fraction of total storage, its impact on working volumes and cycle dynamics—especially in heterogeneous reservoirs—is not negligible. Long-term storage design should quantify adsorbed volumes through high-pressure sorption experiments and include sorption-desorption cycles in reservoir simulation to accurately predict deliverable hydrogen and retention losses.

### 3.3. Biogeochemical Reactions of Hydrogen in Porous Media

In deep saline aquifers, the injection and storage of hydrogen initiate a complex suite of biogeochemical reactions spanning microbial metabolism, redox chemistry, mineral alteration, and gas–water interactions. These processes have profound implications for hydrogen retention, purity, reservoir integrity, and long-term storage reliability.

Microbial activity poses the most significant risk to stored hydrogen. Subsurface environments often host hydrogenotrophic communities, including sulfate-reducing bacteria (SRB), methanogens, and iron-reducing bacteria, capable of converting injected H_2_ into methane or hydrogen sulfide under anaerobic conditions. The principal reactions involve hydrogenotrophic methanogenesis:

(2) $${4H}_{2}+{CO}_{2}\to {CH}_{4}+{2H}_{2}O$$

and sulfate reduction:

(3) $${4H}_{2}+{SO}_{4}^{2-}+2H^{+}\to H_{2}S+{4H}_{2}O$$

These pathways may reduce recoverable hydrogen by up to 40–50% within months if microbial proliferation is unchecked, as observed in field tests at gas storage sites and controlled brine reactors. Methane and sulfide production not only diminishes hydrogen yield but introduces toxicity, corrosion, and safety hazards, particularly in downstream infrastructure.

Abiotic reactions, though generally less aggressive, can alter reservoir rock and fluid chemistry. Hydrogen exposure can induce carbonate dissolution and redox-mediated transformations of iron-bearing minerals (e.g., hematite, goethite, pyrite). Under elevated pressure–temperature conditions typical of hydrogen storage (≥5 MPa, 40–60 °C), modeling and experimental studies have shown dissolution/precipitation altering porosity modestly (<5%) but affecting permeability and caprock integrity if reactions concentrate along seals or faults. In carbonate-rich aquifers, prolonged contact with hydrogen-saturated brine may also shift pH toward neutral or slightly alkaline, with implications for mineral stability.

The dissolution of hydrogen itself into formation water, albeit limited (approx. 1–3 mol % dissolution per year), influences buoyancy, diffusion, and gas–water partitioning. Molecular diffusion of H_2_ is sensitive to temperature and salinity, and accurate transport predictions must incorporate these dependencies alongside Henry’s law constants calibrated under reservoir conditions.

Comprehensive predictions of biogeochemical impacts necessitate coupled reactive-transport modeling, integrating microbial kinetics, mineral reaction networks, and fluid flow. Software tools like PHREEQC (version 3.8.0), TOUGHREACT (version 3), and geochemical variants of CMG-GEM (version 2023.10) have been successfully implemented to simulate long-term hydrogen fate. These models capture the interplay between gas phase transformations, aqueous reactions, microbial metabolism, and changes in porosity or permeability over decadal simulations.

The interplay between microbial hydrogen consumption and abiotic geochemistry dictates not only hydrogen recoverability but also the storage site’s geomechanical stability, fluid phase evolution, and long-term safety. Consequently, reservoir screening for hydrogen storage should mandate microbial community profiling, in situ monitoring of redox-sensitive species, carbonate and sulfide mineral content evaluation, and field deployment of reactive transport experiments. Regulatory and operational frameworks must consider microbial mitigation strategies—such as selective biocides, pH management, or alternative cushion-gas selection—to safeguard both storage efficiency and integrity.

Continued multi-year field and core-scale studies are urgently needed to validate these processes and calibrate predictive models. Only by accounting for the full complexity of biogeochemical reactions can hydrogen storage in deep saline aquifers be deployed at scale with confidence.

## 4. Storage Efficiency and Capacity Calculations for Hydrogen in Saline Aquifers

### 4.1. Volumetric Methods for Estimating Hydrogen Storage Capacity

Estimating the storage capacity of hydrogen in porous media such as deep saline aquifers is a critical component in the assessment and planning of geological hydrogen storage. Several methods have been developed for this purpose, with volumetric methods being among the most employed due to their simplicity and reliance on measurable reservoir properties. These methods estimate the theoretical and practical storage capacity by incorporating fundamental geological parameters such as porosity, aquifer thickness, and areal extent.

The volumetric method calculates storage capacity based on the fundamental Equation for volumetric capacity estimation is expressed as follows:

(4) V= Φ ×h ×A ×S 

where *Φ* is the effective porosity, *h* is the formation thickness, *A* is the areal extent of the aquifer, and *S* represents a saturation term that accounts for the proportion of the pore space that can be occupied by hydrogen under given reservoir conditions [49,50]. This method assumes homogeneous and isotropic conditions and does not directly account for dynamic processes such as fluid flow, capillary trapping, or hysteresis, which are typically handled in more advanced numerical simulations.

Hydrogen storage in deep saline aquifers often draws analogies from CO_2_ geological storage due to similarities in gas behavior, subsurface trapping mechanisms, and reservoir requirements [15,51]. However, hydrogen’s lower molecular weight, higher diffusivity, and greater potential for microbial interaction pose unique challenges that must be considered when applying volumetric estimates [52]. Porosity and permeability measurements, typically derived from well logs, core samples, and seismic data, are foundational to volumetric calculations, while pressure-volume-temperature (PVT) relationships and hydrogen compressibility factors must be carefully integrated to account for real-gas behavior under reservoir conditions [53,54].

Volumetric methods are often used to estimate three levels of storage capacity: theoretical, effective, and practical. Theoretical capacity assumes the entire pore space is available for storage, effective capacity incorporates reservoir constraints such as trapping mechanisms and the irreducible water saturation, while practical capacity accounts for operational limits, including cushion gas requirements and injectivity [55]. These distinctions are critical, especially for hydrogen, where gas recovery is influenced by the relative permeability of gas and water phases, and where cushion gas can play a significant role in maintaining pressure and flow dynamics [56,57].

Recent studies emphasize the importance of incorporating capillary pressure and relative permeability hysteresis into volumetric models, as these factors significantly impact gas trapping and recoverability [58]. Neglecting hysteresis may lead to overestimation of the recoverable hydrogen fraction, particularly in formations with high residual water saturation. Moreover, the choice and behavior of cushion gases, such as nitrogen or natural gas, used to displace hydrogen, can further modify the practical storage capacity and must be factored into advanced volumetric assessments [59].

To enhance the reliability of volumetric methods, they are increasingly coupled with reservoir simulation models that incorporate multiphase flow, microbial interactions, and geomechanical effects. This integration allows for scenario-based evaluations and optimization of storage strategies under varying operational and geological conditions [50]. While volumetric methods remain a foundational approach for preliminary assessments, their predictive potential is significantly improved when supported by site-specific data and validated through dynamic modeling.

### 4.2. Dynamic Modeling and Simulation of Hydrogen Storage

Dynamic modeling has emerged as an indispensable tool in the quantitative assessment and optimization of hydrogen storage in deep saline aquifers. While volumetric methods offer preliminary estimates of storage capacity based on static geological parameters, they are limited in their ability to capture time-dependent processes such as gas migration, multiphase flow interactions, cyclic injection and withdrawal, and chemical or biological transformations. Dynamic simulations provide a more detailed and accurate framework, enabling scenario-based evaluations and informed design of subsurface hydrogen storage systems.

#### 4.2.1. Fundamentals of Dynamic Reservoir Modeling

Dynamic models are typically built on the foundation of multiphase flow theory, employing coupled mass and momentum conservation equations that describe the behavior of hydrogen and brine within porous media. These models simulate hydrogen injection, migration, entrapment, and withdrawal under varying geological and operational conditions. The governing equations account for key parameters such as effective porosity, permeability anisotropy, fluid saturations, capillary pressure, and relative permeability, and are often solved using commercial or open-source reservoir simulators adapted from carbon capture and storage (CCS) applications [50,56].

The unique properties of hydrogen, defined by its low molecular weight, high diffusivity, and low viscosity, require specific adjustments to simulation frameworks originally designed for denser gases like CO_2_. These include the use of real-gas equations of state, precise PVT (pressure-volume-temperature) correlations, and enhanced modeling of capillary forces and hydrogen solubility in brine [60,61]. Key parameters commonly used in dynamic simulations are summarized in Table 2.

#### 4.2.2. Modeling Multiphase Flow and Trapping Mechanisms

In order to simulate in situ hydrogen generation, Okere et al. [62] created a CMG-STARS thermal reservoir model that incorporates coupled mass and energy balance equations. Their model made the assumptions of homogeneous reservoir layers, equilibrium phase interactions, and constant fluid properties; simplifications that successfully lower computational complexity without sacrificing physical accuracy. Without requiring in-depth reactive mineral coupling in initial analyses, a similar strategy can be modified for hydrogen storage modeling to concentrate on multiphase flow and gas migration processes.

One of the principal advantages of dynamic simulation is the ability to model multiphase flow processes, particularly those governing capillary trapping and phase interference. When hydrogen is injected into a saturated formation, it displaces brine and forms a mobile plume that rises due to buoyancy. However, significant portions of hydrogen may become immobilized by residual trapping, governed by capillary pressure hysteresis and pore-throat geometry [63].

These mechanisms are highly sensitive to formation heterogeneity. High-resolution models that include spatially variable permeability and pore structure more accurately predict hydrogen plume dynamics, sweep efficiency, and ultimate recoverability. Pore-scale simulations and micromodel experiments have further demonstrated that viscous fingering, snap-off events, and interfacial tension effects significantly influence the distribution and entrapment of hydrogen in heterogeneous systems [64,65].

#### 4.2.3. Cyclic Injection and Withdrawal: Temporal Dynamics

Dynamic models are especially valuable for simulating cyclical operations, such as those expected in seasonal energy storage. These simulations track the performance of the reservoir across multiple hydrogen injection and production cycles, accounting for changes in reservoir pressure, residual gas saturation, and gas mobility over time [63]. Incorporating temporal dynamics enables accurate estimation of operational storage capacity and deliverability (key metrics that are not captured in static models).

Monte Carlo simulations and sensitivity analyses are increasingly employed to quantify uncertainty in critical parameters, such as formation permeability, capillary entry pressure, and irreducible water saturation. These approaches support probabilistic risk assessments and scenario optimization under geological uncertainty [56].

#### 4.2.4. Cushion Gas Effects and Multicomponent Flow

A key operational consideration in dynamic modeling is the inclusion of cushion gas, an inert or low-reactivity gas that remains in the reservoir to maintain pressure and facilitate hydrogen withdrawal. Common candidates include nitrogen, methane, and carbon dioxide. The behavior of cushion gas significantly impacts gas-phase interactions, relative permeability, and recovery efficiency [66,67]. Additionally, based on simulation results of [68] found that air injection (21% O_2_ + 79% N_2_) produced higher hydrogen yields (≈12 vol%) than pure oxygen or CO_2_ injection because it improved reaction equilibrium and combustion front temperature control. In order to maximize hydrogen-to-syngas ratios and minimize hydrogen losses, the authors determined that an O_2_:N_2_ ratio of 61:39 was ideal. Conceptually, this equilibrium between oxidant dilution and reaction efficiency is similar to cushion gas optimization in underground hydrogen storage, where hydrogen recovery and purity are controlled by the makeup of injected gas mixtures (H_2_ + N_2_/CH_4_/CO_2_) over several injection–withdrawal cycles.

Multicomponent flow models simulate the interactions between hydrogen and cushion gas, accounting for differences in molecular diffusivity, miscibility, and competitive phase occupancy. Research has demonstrated that plume stability and hydrogen recovery efficiency are significantly impacted by gas composition. The work by [38], for example, showed that by stabilizing the gas–water interface and minimizing fingering effects, methane and nitrogen cushions can greatly improve recovery. Similarly, the work by [67] emphasized the significance of gas solubility and diffusivity in determining long-term purity and deliverability, while [66] reported that cushion gas selection regulates plume spreading and gas mixing losses through density and viscosity contrasts. By reducing buoyancy-driven segregation during cyclic storage, optimized gas ratios can enhance injectivity and operational efficiency, as further highlighted by [50,55]. These models are essential for optimizing gas compositions, injection protocols, and withdrawal strategies, particularly in reservoirs where complete hydrogen recovery is economically critical.

#### 4.2.5. Geochemical and Microbial Interactions

Hydrogen’s reactivity introduces potential geochemical and microbial interactions that can influence long-term storage performance. For example, microbial activity may lead to hydrogen consumption through methanogenesis or sulfate reduction, while geochemical reactions may cause mineral dissolution or precipitation, altering porosity and permeability [52].

Reactive transport models, coupled with flow simulations, are increasingly used to evaluate these interactions. Such models can predict zones of microbial dominance, the fate of dissolved hydrogen, and long-term impacts on reservoir integrity, further refining storage capacity estimates [69,70].

#### 4.2.6. Simulation Tools and Case Applications

**Figure 5:**
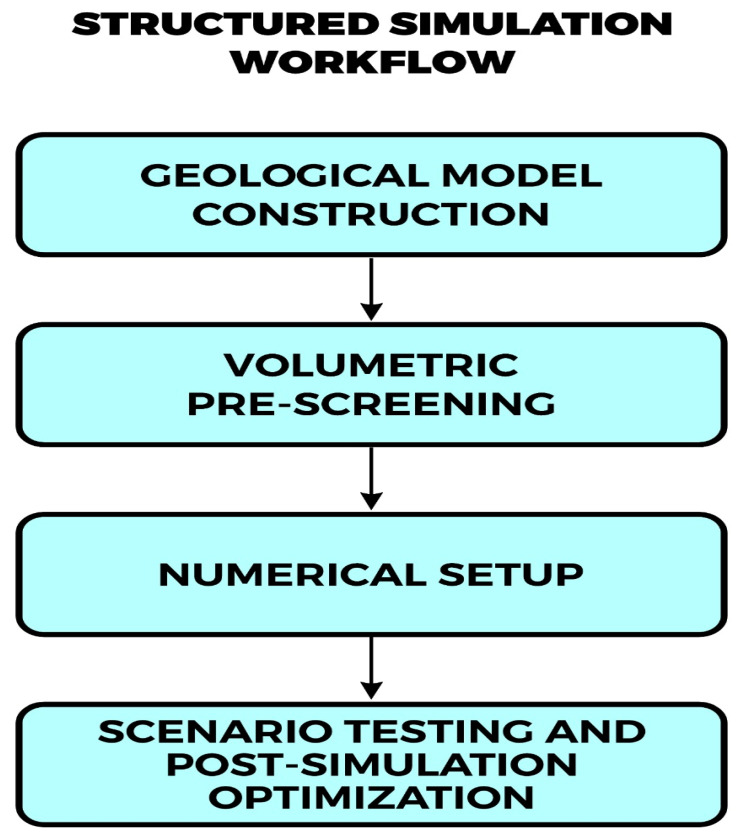
Typical workflow for hydrogen storage simulation and evaluation across various storage media.

A range of simulation platforms has been adapted or developed for hydrogen storage, including TOUGH2, CMG-GEM, and Eclipse, as well as specialized tools like the SafeInCave simulator for salt caverns [71] and the GeoH_2_ Salt Storage and Cycling App [72]. While some tools are tailored to specific storage media (e.g., salt caverns vs. porous formations), their capabilities are converging through modular integration of geomechanical, thermodynamic, and geochemical processes (Figure 5).

These tools generally follow a structured simulation workflow, beginning with geological model construction, followed by volumetric pre-screening, numerical setup, scenario testing, and post-simulation optimization. A conceptual outline of this process is illustrated in Figure 5, emphasizing the integration of reservoir data, operational constraints, and dynamic performance metrics.

#### 4.2.7. Summary and Outlook

Dynamic modeling provides a comprehensive framework for understanding the behavior of hydrogen in deep saline aquifers under real-world operating conditions. It bridges the gap between theoretical storage potential and practical, recoverable capacity by accounting for multiphase flow, geological heterogeneity, gas interactions, and operational cycling. When combined with static volumetric methods, laboratory data, and field observations, dynamic simulation forms the cornerstone of rigorous, site-specific storage assessments.

Continued development of multiphysics simulators, coupled with advances in data integration, will enhance the predictive capability of these models. Future work should focus on standardizing simulation practices, improving representation of microbial and geochemical effects, and validating model outcomes through pilot-scale field deployments.

### 4.3. Practical Considerations in Storage Capacity Estimates

While theoretical calculations provide a baseline for estimating hydrogen storage capacity in deep saline aquifers, translating these estimates into deployable projects demands a detailed understanding of several practical constraints. These factors ranging from subsurface geological complexity to surface-level operational and economic challenges can significantly reduce the amount of hydrogen that can be effectively stored and cycled over time. Table 3 provides a summary of key considerations and their potential impact on capacity evaluations.

Although theoretical models may suggest substantial hydrogen storage potential in saline aquifers, the combined influence of these practical considerations substantially narrows the realizable capacity [41,83]. Geological heterogeneity leads to preferential flow paths and gas bypassing, reducing efficiency and complicating reservoir management. Cushion gas demands are particularly significant, often occupying most of the usable pore volume, sometimes over two-thirds, thus reducing the effective storage capacity. Wettability and hysteresis further restrict mobility and recovery by enhancing capillary trapping, especially under cyclic operations. Operational limitations, such as pressure constraints and wellbore integrity, restrict both injection rates and reservoir accessibility. Finally, economic considerations often dictate whether technically viable projects proceed, especially when infrastructure, cushion gas costs, and monitoring expenses are considered. Therefore, any comprehensive storage assessment must integrate these interrelated factors to avoid overestimation and to design viable, resilient storage systems.

### 4.4. Pressure and Time Effect on Storage Efficiency in Deep Saline Aquifers

The efficiency of hydrogen storage in deep saline aquifers is significantly influenced by operational parameters such as pressure and time. These factors affect various physical and chemical processes, including gas dissolution, capillary trapping, hysteresis, and microbial activity, all of which impact the recoverability and purity of stored hydrogen.

#### 4.4.1. Pressure Dynamics and Hydrogen Recovery

**Figure 6:**
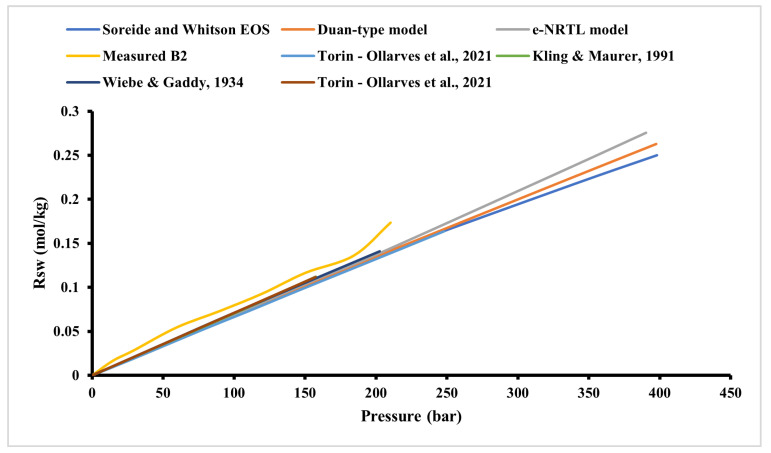
Hydrogen solubility in brine showing dissolution rates as pressure increases (modified from [26,84]). Data from Soreide and Whitson EOS [85], Duan-type model [86], e-NRTL model [87], Measured B2 [84], Torin-Ollarves et al., [88], Kling & Maurer [89], and Wiebe & Gaddy [90].

Injection pressure is a critical determinant of hydrogen storage efficiency. Elevated pressures can enhance hydrogen solubility in the brine, leading to increased dissolution losses. Experimental studies have shown that hydrogen solubility in the brine is pressure-dependent, with higher pressures resulting in greater dissolution rates. This dissolved hydrogen becomes challenging to recover, thereby reducing overall storage efficiency (Figure 6).

Moreover, pressure influences capillary trapping mechanisms. Higher injection pressures can lead to increased capillary trapping of hydrogen within the pore spaces of the reservoir rock. This trapped hydrogen is not readily recoverable during withdrawal phases, further diminishing storage efficiency.

#### 4.4.2. Temporal Effects and Cyclic Operations

The duration of hydrogen storage and the frequency of injection-withdrawal cycles also play crucial roles in determining storage efficiency. Over extended periods, hydrogen can undergo microbial consumption and chemical reactions within the reservoir. Microbial activity, particularly from sulfate-reducing bacteria and methanogens, can consume hydrogen, leading to the production of hydrogen sulfide and methane, which contaminate the stored hydrogen and reduce its purity [91,92].

Additionally, repeated cycling can induce hysteresis effects in the reservoir’s relative permeability and capillary pressure characteristics. These hysteresis effects can lead to increased trapping of hydrogen and reduced mobility, thereby decreasing the amount of hydrogen that can be recovered during withdrawal phases.

#### 4.4.3. Operational Strategies for Enhanced Efficiency

To mitigate the adverse effects of pressure and time on storage efficiency, several operational strategies can be employed:

➢ Optimizing injection pressure: Maintaining injection pressures within an optimal range can minimize hydrogen dissolution and capillary trapping.
➢ Managing storage duration: Limiting the duration of hydrogen storage can reduce the extent of microbial consumption and chemical reactions that degrade hydrogen purity.
➢ Implementing cushion gases: Using inert cushion gases like nitrogen or methane can help maintain reservoir pressure and reduce hydrogen losses during cycling operations.
➢ Monitoring and controlling microbial activity: Employing biocides or other microbial control measures can mitigate the consumption of hydrogen by subsurface microorganisms.

In conclusion, understanding and managing the effects of pressure and time are crucial for optimizing hydrogen storage efficiency in deep saline aquifers. By implementing appropriate operational strategies, it is possible to enhance hydrogen recoverability and maintain the quality of stored hydrogen over time.

## 5. Factors Affecting Hydrogen Injection and Plume Evolution in Deep Saline Aquifers

The successful implementation of underground hydrogen storage in deep saline aquifers depends on a detailed understanding of the physical, chemical, and biological processes that govern hydrogen behavior post-injection. These processes influence not only the storage capacity and efficiency but also the retrievability and purity of the stored hydrogen. Key factors include reservoir heterogeneity, fluid properties, capillary and viscous forces, geochemical interactions, and microbial activity. This section synthesizes current knowledge on these aspects, highlighting their implications for hydrogen plume dynamics and storage performance.

### 5.1. Technical and Scientific Challenges Saline Aquifer Storage

#### 5.1.1. Multiphase Flow Dynamics and Fingering Phenomena

Hydrogen injection into saline aquifers initiates complex multiphase flow dynamics, primarily due to the significant contrast in viscosity and density between hydrogen and resident brine. This disparity often leads to unstable displacement patterns, such as viscous and capillary fingering, which can result in uneven hydrogen distribution and entrapment in less accessible zones of the reservoir. Experimental studies employing core flooding and imaging techniques have demonstrated that such fingering effects are pronounced in heterogeneous formations, leading to reduced sweep efficiency and lower hydrogen recoverability [78].

#### 5.1.2. Capillary Trapping and Relative Permeability Hysteresis

**Figure 7:**
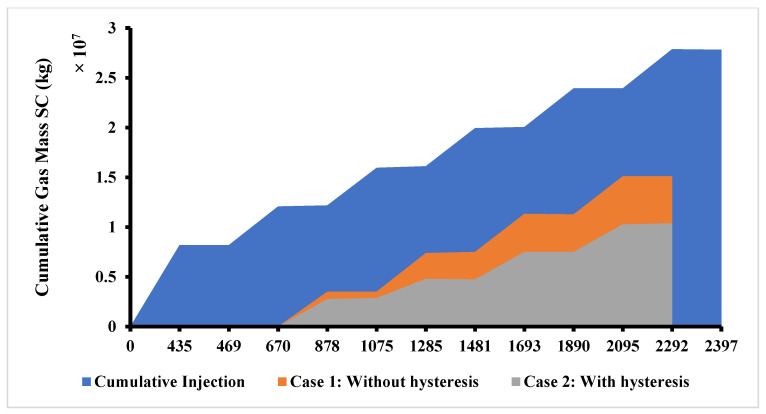
Cumulative injection showing effects of capillary trapping and permeability hysteresis (modified from [38]).

Capillary forces play a critical role in hydrogen entrapment within pore spaces, particularly during the withdrawal phase. The phenomenon of relative permeability hysteresis, where the path of fluid saturation during imbibition differs from drainage, further complicates hydrogen recovery. Numerical simulations have indicated that hysteresis effects can significantly diminish hydrogen recovery rates, with some studies reporting reductions from 78% to 45% over multiple injection-withdrawal cycles (Figure 7). These findings underscore the necessity of incorporating hysteresis models into reservoir simulations to accurately predict storage performance.

#### 5.1.3. Geochemical Interactions and Mineral Alteration

Hydrogen’s reactivity poses challenges related to geochemical interactions within the reservoir. While hydrogen is relatively inert under standard conditions, its presence can influence redox reactions, potentially leading to mineral dissolution or precipitation. For instance, interactions with iron-bearing minerals may result in the formation of hydrogen sulfide, compromising the purity of the stored gas. Moreover, changes in mineralogy can alter porosity and permeability, affecting the reservoir’s storage characteristics.

#### 5.1.4. Microbial Activity and Biogeochemical Transformations

Microbial communities present in saline aquifers can metabolize hydrogen, leading to its consumption and the production of byproducts such as methane and hydrogen sulfide. These biogeochemical transformations not only reduce the volume of recoverable hydrogen but also pose risks related to gas quality and infrastructure integrity. Biofilm formation can clog pore spaces, decreasing permeability, while microbial-induced corrosion can damage well casings and surface facilities [78]. Mitigating these effects requires a comprehensive understanding of the subsurface microbiome and the implementation of monitoring and control strategies.

#### 5.1.5. Cushion Gas Dynamics and Gas Mixing

**Figure 8:**
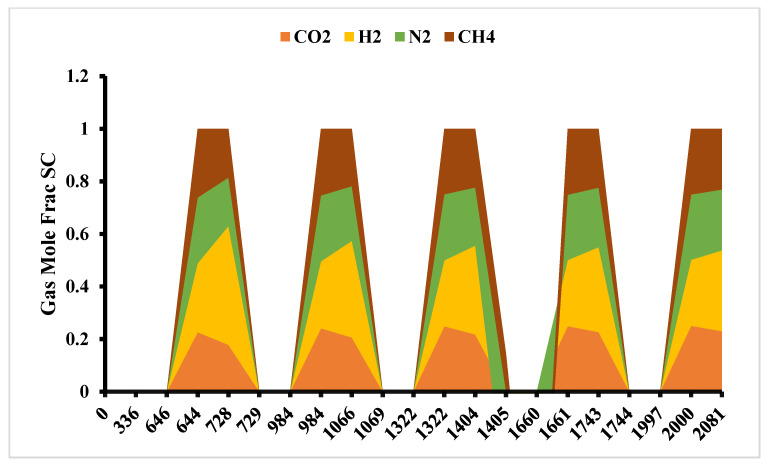
Effect of different cushion gases based on balancing between storage efficiency and product quality (modified from [93]).

The use of cushion gases, such as nitrogen or methane, is essential to maintain reservoir pressure and facilitate hydrogen withdrawal. However, the mixing of hydrogen with cushion gases can lead to dilution, affecting the purity of the extracted hydrogen. Studies have shown that while methane serves as an effective cushion gas in minimizing hydrogen loss, it may compromise gas purity (Figure 8). Therefore, selecting an appropriate cushion gas involves balancing the trade-offs between storage efficiency and product quality.

#### 5.1.6. Induced Seismicity and Geomechanical Considerations

Injecting hydrogen into deep formations alters the in situ stress regime, potentially triggering seismic events, especially in tectonically active regions. While the risk of induced seismicity in saline aquifers is generally lower compared to other formations, it remains a concern that necessitates thorough geomechanical assessments and continuous monitoring [94]. Understanding the mechanical behavior of the reservoir rock under cyclic loading conditions is crucial to ensure the long-term stability and safety of the storage operation.

### 5.2. Boundary Conditions in Hydrogen Injection and Plume Evolution in Deep Saline Aquifers

Boundary conditions play a critical role in determining the behavior of hydrogen during injection, migration, and withdrawal in deep saline aquifers. These conditions include the physical limits of the storage formation, the geological and hydrodynamic constraints imposed by surrounding strata, and the operational parameters defined by injection strategies. Together, they shape how hydrogen distributes within the reservoir, how much is trapped or lost, and ultimately, the efficiency and capacity of the storage system.

From a geological perspective, the nature of the reservoir boundaries, particularly the presence and integrity of overlying caprocks governs vertical containment. Caprocks composed of shale or evaporites are typically characterized by low permeability and high capillary pressures, providing a primary seal against hydrogen escape. Structural features such as faults and folds can either enhance storage by forming traps or compromise containment if they are transmissive or reactivated under increased injection pressures. Geological heterogeneities within the reservoir, including lateral variations in porosity and permeability, create preferential flow paths that significantly influence plume geometry and sweep efficiency. These heterogeneities can lead to early hydrogen breakthrough, poor areal coverage, and reduced recoverability.

Hydrodynamic conditions, such as ambient reservoir pressure, temperature, and the salinity of the formation brine, also shape the behavior of the hydrogen plume. Hydrogen’s low density and viscosity relative to brine promotes buoyant and viscous fingering during injection, increasing the likelihood of uneven displacement and bypassed zones. Elevated reservoir pressures can increase hydrogen solubility in brine, leading to storage losses through dissolution, whereas insufficient pressure may limit injectivity. The direction and magnitude of regional groundwater flow can either assist plume containment or promote lateral migration away from the injection zone, complicating storage control and retrieval.

Operationally, the way hydrogen is injected and withdrawn cycle frequency, flow rates, and cumulative volumes has a direct effect on the pressure regime within the storage formation. Cyclic injections can induce pressure oscillations that impact capillary trapping and residual gas saturation. If pressure build-up exceeds the fracture gradient of the caprock, mechanical failure and potential leakage pathways may form. To mitigate this, cushion gases such as nitrogen or methane are often used to stabilize pressure and support hydrogen mobility. However, mixing between cushion gas and hydrogen may reduce product purity upon withdrawal, presenting a trade-off between injectivity and gas quality. The choice of cushion gas should therefore be based on site-specific compatibility, miscibility, and operational goals.

Microbial and geochemical boundary conditions further influence long-term storage performance. Hydrogen can serve as an electron donor for subsurface microorganisms, particularly sulfate-reducing bacteria and methanogens, which consume hydrogen and generate byproducts such as hydrogen sulfide and methane. These reactions not only reduce the volume of recoverable hydrogen but may also alter reservoir geochemistry and damage infrastructure through corrosion. In addition, redox reactions between hydrogen and iron-bearing minerals can cause porosity changes or mineral precipitation, affecting injectivity and flow pathways. These processes are slow but important, especially for long-duration storage scenarios, and should be incorporated into reservoir models when assessing site suitability.

Finally, accurate representation of boundary conditions in numerical simulations is essential for predicting hydrogen plume evolution and optimizing storage strategies. Boundary conditions define the extent of the model domain and control how fluids enter and leave the system. Incorrect assumptions such as imposing impermeable lateral boundaries in an open aquifer system can lead to erroneous predictions of plume migration, pressure buildup, and hydrogen recovery. Recent studies advocate for high-resolution site characterization and stochastic modeling to account for uncertainties in boundary geometry, permeability distribution, and caprock sealing capacity. When integrated into coupled reactive transport and multiphase flow simulations, such models provide a more robust framework for assessing storage efficiency and containment risks under realistic geological scenarios.

In summary, boundary conditions, geological, hydrodynamic, and operational are not merely modeling parameters but fundamental controls on hydrogen behavior in subsurface environments. A detailed understanding and accurate incorporation of these conditions are essential to achieve reliable estimates of storage efficiency and to design safe, scalable hydrogen storage projects in deep saline aquifers.

### 5.3. Driving Forces and Fluid Properties

The behavior of hydrogen during injection and storage in deep saline aquifers is governed by a complex interplay of driving forces and fluid properties. Understanding these factors is crucial for optimizing storage efficiency, ensuring containment, and maximizing recoverability.

For example, hydrogen generation and migration efficiency are greatly impacted by reservoir properties, especially porosity, permeability, and temperature. According to Okere et al. [62] temperature has a significant influence on reaction kinetics, while porosity and permeability determine gas distribution and combustion efficiency. Their results also demonstrated that CH_4_–CO_2_ mixtures have synergistic effects through gasification and reforming reactions, increasing hydrogen yield. This mechanism is conceptually similar to the reactive transport phenomena and multiphase flow that controls the behavior of hydrogen storage in deep saline aquifers.

Hydrogen’s low density relative to brine results in strong buoyant forces that drive its upward migration within the reservoir. This buoyancy can lead to gravity override, where hydrogen accumulates at the top of the formation, potentially causing uneven distribution and early breakthrough during withdrawal. The extent of this phenomenon is influenced by reservoir heterogeneity and the presence of structural traps.

Hydrogen’s low viscosity compared to brine contributes to high mobility, which can exacerbate viscous fingering during injection. This instability leads to inefficient displacement of brine, resulting in reduced sweep efficiency and increased residual brine saturation. The mobility ratio, defined as the ratio of hydrogen mobility to brine mobility, is a key parameter in predicting the onset and severity of fingering phenomena.

The interfacial tension between hydrogen and brine affects capillary forces within the porous media. High interfacial tension can lead to increased capillary pressure, influencing the distribution and trapping of hydrogen. Capillary forces also play a role in snap-off events, where hydrogen becomes disconnected and trapped in pore spaces, reducing recoverability.

The wettability of the reservoir rock determines the preferential flow paths of fluids. In water-wet systems, brine tends to occupy smaller pores, while hydrogen occupies larger pores. This distribution affects relative permeability and the ease with which hydrogen can be injected and withdrawn. Studies have shown that wettability conditions significantly impact hydrogen recovery, with water-wet conditions generally favoring higher recoverability.

The relative permeability of hydrogen and brine dictates the ease of fluid flow through the reservoir. Hysteresis effects, arising from differences in drainage and imbibition cycles, can lead to residual trapping of hydrogen and reduced recovery during withdrawal. Accounting for hysteresis in reservoir simulations is essential for accurate prediction of storage performance.

Hydrogen’s solubility in brine, though relatively low, can lead to dissolution losses over time. Factors such as pressure, temperature, and brine salinity influence solubility levels. Higher pressures and lower salinities increase hydrogen solubility, potentially leading to greater losses. Understanding these dynamics is important for long-term storage planning.

Molecular diffusion and mechanical dispersion contribute to the spreading of the hydrogen plume within the reservoir. While diffusion is generally slow, over extended periods, it can lead to significant spreading and dilution of the hydrogen, affecting both containment and purity. Dispersion, influenced by reservoir heterogeneity and flow rates, can also impact plume evolution.

The presence of hydrogenotrophic microorganisms in the reservoir can lead to biological consumption of hydrogen, producing byproducts such as methane and hydrogen sulfide. This microbial activity not only reduces the volume of recoverable hydrogen but also poses risks to gas quality and infrastructure integrity.

Hydrogen can participate in redox reactions with minerals in the reservoir, potentially altering porosity and permeability. These reactions may also impact the long-term stability and integrity of the storage site. Understanding the geochemical interactions between hydrogen, brine, and reservoir minerals is essential for assessing storage feasibility.

### 5.4. Hydrogen-Water Displacement Behavior and Its Impact on Storage Efficiency

The displacement behavior of hydrogen in sedimentary formations is governed by a complex interplay of multiphase flow dynamics, pore-scale heterogeneity, and fluid-rock interactions. Understanding these characteristics is crucial for optimizing storage efficiency and ensuring the integrity of underground hydrogen storage systems in deep saline aquifers [95].

**Figure 9:**
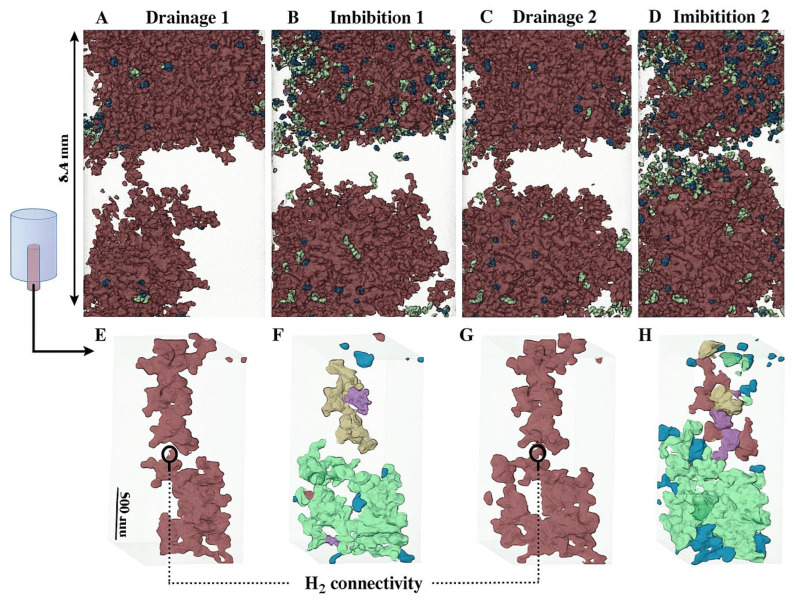
Pore scale heterogeneity in reservoir showing hydrogen distribution and trapping mechanisms in drainage and imbibition. The distribution of the H_2_ phase throughout the entire rock sample is visualized after each stage of H_2_ displacement: (**A**) first drainage, (**B**) first imbibition, (**C**) second drainage, and (**D**) second imbibition. Green indicates that H_2_ occupy pores between 10 and 100 times the average size, blue indicates that H_2_ occupy pores up to 10 times the average size, and brown indicates that connected H_2_ occupy pore volumes more than 100 times the average pore size in panels (**A**–**D**). Each displacement step is followed by additional visualizations for a subsection of the rock sample: panels (**E**,**G**) depict the smallest throats that facilitate H_2_ connectivity during the first and second drainages, respectively, while panels (**F**,**H**) show isolated, disconnected H_2_ ganglia that remain after the first and second imbibition stages [95] (modified from [95]).

Recent studies employing high-resolution X-ray imaging have revealed that hydrogen tends to preferentially occupy larger pores and bypass smaller ones during injection, leading to inefficient sweep and reduced storage capacity (Figure 9). This behavior is influenced by the non-wetting nature of hydrogen relative to brine, resulting in capillary forces that hinder its entry into finer pore spaces. Such dynamics underscore the importance of accounting for pore-scale heterogeneity in reservoir models to accurately predict hydrogen distribution and trapping mechanisms.

Experimental measurements have demonstrated that hydrogen exhibits lower relative permeability compared to other gases like methane and nitrogen in fractured carbonate rocks. This reduced permeability is attributed to intermittent fluid connectivity and phase interference, which are not adequately captured by traditional relative permeability models. Moreover, capillary pressure curves indicate that hydrogen requires higher entry pressures to displace brine, further complicating its migration through the reservoir.

The cyclic nature of hydrogen injection and withdrawal introduces hysteresis in relative permeability and capillary pressure relationships. During imbibition (brine re-entry), residual hydrogen trapping occurs, leading to irreversible losses and decreased recoverability. This hysteresis effect is exacerbated by the wettability characteristics of the reservoir rock and the interfacial tension between hydrogen and brine.

Small-scale heterogeneities, such as layering and variations in pore throat sizes, significantly influence hydrogen displacement patterns. These heterogeneities can cause uneven hydrogen distribution, preferential flow paths, and localized trapping, which are often overlooked in large-scale reservoir simulations. Incorporating detailed geological features into modeling efforts is essential for accurate prediction of hydrogen behavior and storage performance.

The aforementioned displacement characteristics have direct implications for the efficiency and capacity of hydrogen storage in sedimentary formations. Inefficient sweep, residual trapping, and hysteresis effects can lead to substantial hydrogen losses and reduced deliverability. To mitigate these challenges, strategies such as optimizing injection rates, employing cushion gases, and enhancing reservoir characterization are recommended.

In summary, the displacement behavior of hydrogen in sedimentary rocks is influenced by a multitude of factors, including pore-scale dynamics, fluid properties, and reservoir heterogeneity. A comprehensive understanding of these elements is vital for the successful implementation of underground hydrogen storage in deep saline aquifers.

### 5.5. Comparative Analysis of Factors Influencing Hydrogen Storage in Deep Saline Aquifers

To synthesize the various technical, geological, and operational aspects discussed, Table 4 provides a comparative overview of the key factors influencing hydrogen storage dynamics in deep saline aquifers. Each factor is assessed in terms of its origin, effect on storage performance, and implications for modeling and operational design.

Table 4 reveals that while all factors influence hydrogen storage, their mechanisms of action and operational consequences vary significantly. Fluid-driven instabilities such as multiphase flow behavior and buoyancy primarily govern initial hydrogen distribution and are most sensitive to reservoir heterogeneity and injection strategy. In contrast, capillary trapping and hysteresis emerge during withdrawal cycles, influencing long-term recoverability and requiring more detailed modeling of saturation history. Geochemical and microbial factors, though slower-acting, present persistent threats to gas purity and system integrity, underscoring the need for reactive transport and bio-geochemical monitoring. Operational considerations like cushion gas selection and boundary conditions cut across the injection-withdrawal cycle, introducing both design flexibility and complexity. Finally, induced seismicity, though less commonly emphasized, could pose significant regulatory and safety concerns, especially in tectonically sensitive areas. Collectively, these comparisons illustrate that a holistic, coupled-process modeling approach incorporating geological, hydrodynamic, chemical, and biological interactions is essential for designing robust underground hydrogen storage systems in saline aquifers.

## 6. Comparative Summary of Key Findings in the Recent Literature

Although the processes governing hydrogen storage are described in the preceding sections, it is equally crucial to synthesize quantitative insights from previous studies in order to better understand their relative significance. Key parameters from recent experimental, modeling, and economic evaluations are compiled in Table 5. These include levelized cost of storage (LCOS), microbial consumption rates, and hydrogen recovery efficiencies. The comparative data show significant variation between studies, with microbial hydrogen consumption ranging from 10 to 50%, hydrogen recovery ranging from 45 to 85%, and LCOS values ranging from 1.3 to 3.4 USD kg^−1^. These variations reflect variations in reservoir heterogeneity, cushion gas selection, and operational design. These quantitative comparisons highlight how geochemical-microbial activity and reservoir-scale multiphase flow behavior interact to most strongly control storage performance, while recovery efficiency and cushion gas optimization play a major role in economic viability.

This review goes beyond descriptive analysis by combining these quantitative results into a critical synthesis that pinpoints the biological and operational thresholds governing the cost and efficiency of hydrogen storage in deep saline aquifers.

## 7. Economics of Hydrogen Storage in Deep Saline Aquifers

As hydrogen emerges as a key vector in decarbonizing energy systems, the need for large-scale, flexible, and geographically distributed storage becomes increasingly urgent. Among the storage options available, deep saline aquifers offer the largest theoretical capacity and geographic flexibility. However, their development is still in its infancy compared to mature technologies like salt caverns. This chapter explores the economic, techno-strategic, and policy dimensions of aquifer-based hydrogen storage. It builds on the technical foundation laid in previous chapters and presents an integrated view of the key drivers, barriers, and opportunities for deployment.

### 7.1. Costs Associated with Aquifer-Based Hydrogen Storage

Storing hydrogen in deep saline aquifers offers a promising solution for large-scale, long-duration energy storage, particularly to buffer seasonal fluctuations in renewable electricity generation. However, the economic feasibility of this approach depends on a combination of interrelated cost factors, including capital expenditures, operational costs, and the significant requirement for cushion gas.

#### 7.1.1. Capital Expenditures

Capital costs for developing hydrogen storage infrastructure in saline aquifers are generally higher than for other subsurface storage options. A dominant component of these costs often exceeding 80%, is the requirement for cushion gas, which serves to maintain reservoir pressure and ensure efficient recovery of injected hydrogen. By contrast, in salt cavern storage, capital expenditures are primarily driven by site preparation and solution mining. A techno-economic analysis focusing on the U.S. Intermountain West estimated capital costs of approximately $22.4 per kilogram of stored hydrogen for saline aquifers, compared to $15.0 for salt caverns and $6.2 for depleted gas reservoirs [37,96,97].

#### 7.1.2. Operational Expenses

Operational expenses encompass compression, injection, monitoring, and routine maintenance activities. These costs are influenced by formation depth, pressure and temperature conditions, and cycling frequency [37]. For saline aquifers, operational costs tend to be higher relative to other geologic formations due to the added complexity of managing multiphase flow, hydrogen-brine interactions, and the potential for geochemical reactivity with host rock minerals [37].

#### 7.1.3. Cushion Gas Requirements

Cushion gas is critical for maintaining reservoir pressure and establishing favorable flow conditions for hydrogen withdrawal. In saline aquifers, cushion gas volumes can represent up to 80% of the total gas inventory in the storage reservoir [96], thereby exerting a significant impact on upfront capital investment. Strategies to mitigate cushion gas costs include the use of inert or less expensive gases, such as nitrogen or natural gas, to partially displace hydrogen as the working gas. Such approaches can lower the overall cost while maintaining injectivity and deliverability, though they may introduce additional operational and compositional complexity.

#### 7.1.4. Comparative Economic Analysis and Outlook

Comparative studies of levelized cost of storage (LCOS) across different geological settings consistently show saline aquifers as the most cost-intensive option. LCOS estimates range from approximately $3.4 per kilogram of hydrogen in saline aquifers to $2.3 for salt caverns and $1.3 for depleted gas fields [96,97]. These disparities are largely attributable to differences in cushion gas requirements, capital intensity, and operational uncertainties.

Nonetheless, several factors may help reduce the cost differential over time. Advances in reservoir modeling, well completion techniques, and cushion gas optimization have the potential to improve efficiency and reduce economic barriers. Moreover, scaling effects, market maturation, and policy-driven incentives for long-duration energy storage could further enhance the viability of aquifer-based hydrogen storage. Continued interdisciplinary research and supportive regulatory frameworks will be vital to unlocking the full economic potential of this storage pathway within a decarbonized energy system.

### 7.2. Economic Risk and Sensitivity Analysis

While the preceding sections have presented capital and operational cost estimates for aquifer-based hydrogen storage, the economic feasibility of these systems cannot be fully understood without analyzing the uncertainties that shape project outcomes. Unlike salt caverns and depleted gas fields, saline aquifers involve a higher degree of geological and operational unpredictability, which directly impacts cost estimations and return on investment. Risk and sensitivity analyses serve as essential tools to quantify these uncertainties, guide investment decisions, and inform policy interventions.

Economic risk in aquifer storage projects arises from both technical parameters such as injectivity, cushion gas behavior, and hydrogen recovery efficiency and external market conditions, including electricity prices, hydrogen demand, regulatory shifts, and carbon pricing. For example, cushion gas costs, which constitute the most upfront expenditures, are highly sensitive to gas prices and the assumed retention rate over the project lifecycle. Similarly, hydrogen deliverability efficiency affects not only storage performance but also the LCOS, especially under high cycling frequencies.

To evaluate the influence of these variables, Monte Carlo simulations and probabilistic cash flow models are often employed. These models allow for multiple runs with randomly sampled input values based on assigned probability distributions. For instance, a simulation might model variations in cushion gas cost (±25%), storage efficiency (±15%), and capital cost escalation (±20%) to produce a range of potential net present value (NPV) or LCOS outcomes. The output is typically visualized through a cumulative distribution function (CDF) of LCOS or a tornado diagram showing the variables with the greatest impact on the economic outcome.

A simplified form of the LCOS calculation is given by the following Equation:

(5) $$LCOS=\;\frac{\sum_{t=1}^{n}\left(\frac{C_{cap,t}+C_{op,t}+C_{cg,t}}{{\left(1+r\right)}^{t}}\right)}{\sum_{t=1}^{n}\left(\frac{E_{H_{2}},t}{{\left(1+r\right)}^{t}}\right)}\;$$

where $C_{cap,t}$ is the capital cost in year *t*; $C_{op,t}$ represents the operational and maintenance cost in year *t*; $C_{cg,t}$ cushion gas cost allocated to year *t*; $E_{H_{2}},t$ denotes the quantity of hydrogen delivered in year *t*; *r* represents the discount rate and the project lifespan in years.

Equation (5) highlights how economic returns are closely related to the time value of money, storage efficiency, and hydrogen output, and how any change in input assumptions alters the LCOS.

Table 6 presents a representative output from a sensitivity analysis conducted on a hypothetical saline aquifer hydrogen storage project in the Permian Basin. It details the influence of key parameters on the LCOS, based on a model developed for illustrative purposes. While the data reflects general trends reported in the literature, the analysis does not correspond to any specific published study. Nonetheless, similar studies have been conducted, offering valuable insights into the economic drivers of underground hydrogen storage systems [11,38].

Table 5 shows the disproportionate influence of cushion gas costs and hydrogen recovery efficiency on the overall storage economics. Consequently, targeted research into alternative cushion gases, cyclic optimization strategies, and enhanced well completion techniques offers the greatest potential for reducing economic risk.

Even when hydrogen co-production is technically possible, oil revenue continues to be the primary driver of profitability from a techno-economic perspective. Despite producing less hydrogen, air injection was found to be more cost-effective than CO_2_ + O_2_ injection due to lower capital and recycling costs [98]. The analysis emphasizes that, in the absence of carbon credits or policy incentives, hydrogen production on its own is currently not economically feasible. This is similar to the cost dynamics of storing hydrogen underground, where reservoir quality, injection design, and cushion gas selection all have a significant impact on levelized cost of storage (LCOS).

Moreover, regulatory environments play a crucial role in risk valuation. Uncertainty surrounding long-term site liability, hydrogen purity standards, and permitting timelines can result in increased financial risk premiums or delayed investment. Integration of policy-based risk mitigation such as loan guarantees, tax incentives, or performance-based subsidies can improve investor confidence and shift borderline projects into financially viable territory.

In summary, a comprehensive economic analysis of hydrogen storage in saline aquifers must go beyond static cost comparisons and engage with the full spectrum of financial risk and variability. Sensitivity and probabilistic analyses offer critical insights for project developers, investors, and policymakers by highlighting the most impactful parameters and guiding where innovation or regulatory support can yield the highest returns. As hydrogen markets mature, the ability to transparently and rigorously quantify risk will be as important as reducing costs themselves.

### 7.3. Roadmap for Developing Hydrogen Storage in Aquifers

The transition to a low-carbon energy future depends on establishing scalable and reliable hydrogen storage infrastructure, with deep saline aquifers offering immense potential due to their vast capacity and widespread geographic distribution. However, commercial-scale deployment of hydrogen storage in aquifers requires a phased, multi-disciplinary development strategy that addresses geotechnical uncertainties, regulatory gaps, cost barriers, and public perception. A coherent roadmap drawing on recent initiatives such as the HyUsPRe project and key EU strategic frameworks can provide a structured approach to advance aquifer-based storage from pilot to commercial stages.

This staged approach is summarized in Table 7, which outlines the recommended actions and focus areas across short-, medium-, and long-term horizons. The roadmap emphasizes that scientific innovation must proceed in parallel with regulatory clarity and societal trust to ensure the successful deployment of aquifer-based hydrogen storage.

The initial phase of the roadmap focuses on site screening and characterization, where geologic formations are evaluated based on porosity, permeability, caprock integrity, and geochemical compatibility with hydrogen. During this phase, extensive laboratory and field studies are needed to better understand hydrogen–brine–rock interactions, microbial activity, and potential impacts on reservoir properties. Equally important is the design and execution of small-scale pilot projects to validate containment performance and to build confidence in predictive models. These early efforts are critical to de-risk storage operations and provide empirical data for scaling up.

The intermediate phase prioritizes cost reduction and regulatory alignment. High capital expenditures, particularly due to cushion gas requirements currently limit economic feasibility. Research into cost-effective alternatives, such as using inert or low-value gases for pressure maintenance, could significantly reduce the LCOS. Simultaneously, regulatory frameworks must evolve to accommodate hydrogen-specific safety protocols, risk assessment methodologies, and long-term liability mechanisms. Developing harmonized permitting pathways across jurisdictions will also facilitate investor confidence and cross-border hydrogen trade.

In the final phase, the roadmap envisions commercial deployment and public engagement, where hydrogen storage hubs integrate aquifers with electrolyzer clusters and distribution networks. At this stage, public outreach becomes critical, particularly in addressing concerns related to subsurface integrity, induced seismicity, and environmental monitoring. Transparent data sharing and stakeholder engagement strategies, supported by demonstration-scale projects, are essential to gaining societal acceptance.

## 8. Challenges of Hydrogen Storage in Deep Saline Aquifers

Deep saline aquifers (DSAs) are increasingly recognized for their potential in large-scale underground hydrogen storage (UHS), offering vast capacities and widespread geographic availability. However, several technical and scientific challenges must be addressed to ensure the safe, efficient, and long-term viability of hydrogen storage in these formations.

### 8.1. Geochemical Interactions and Hydrogen Loss

The injection of hydrogen into DSAs initiates complex geochemical reactions between hydrogen, formation brines, and mineral matrices. These reactions can lead to hydrogen consumption through redox processes with minerals such as pyrite (FeS_2_), resulting in the formation of hydrogen sulfide (H_2_S), which degrades gas quality and poses safety hazards. Additionally, hydrogen-induced mineral dissolution or precipitation can alter porosity and permeability, affecting injectivity and potentially compromising caprock integrity. For example, according to Okere and Sheng [101], chemical and biological reactions in the reservoir environment can result in hydrogen losses in in situ reservoir systems. Microbial communities and redox reactions become active as hydrogen moves from hot spots to colder areas, consuming hydrogen and producing byproducts like water and methane. It was discovered that these biogeochemical changes have an impact on overall process efficiency and drastically lower recoverable hydrogen yields.

While some studies suggest that pure geochemical reactions may have minimal impact over short durations, the long-term effects, especially when coupled with microbial activity, require further investigation.

### 8.2. Microbial Activity and Biogeochemical Effects

The introduction of hydrogen into DSAs can stimulate indigenous microbial communities, leading to hydrogen consumption by microorganisms such as methanogens and sulfate-reducing bacteria. These microbes metabolize hydrogen, producing CH_4_ and H_2_S, thereby reducing stored hydrogen volumes and contaminating the gas. Furthermore, microbial growth can lead to biofilm formation, clogging pore spaces and reducing permeability, which impairs gas injectivity and withdrawal efficiency. Understanding and mitigating these biogeochemical processes are essential for maintaining hydrogen purity and storage efficiency.

### 8.3. Hydrodynamic Challenges and Gas Recovery Efficiency

Hydrogen’s low viscosity and high mobility relative to formation brines can result in viscous fingering and gravity override, leading to uneven gas displacement, reduced storage efficiency, and complications during withdrawal operations. Capillary forces can trap hydrogen in pore spaces, leading to significant residual gas saturation and reduced recoverability. Neglecting hysteresis effects in modeling can result in substantial errors in estimating recoverable hydrogen volumes.

### 8.4. Geomechanical Stability and Caprock Integrity

The cyclical injection and withdrawal of hydrogen can induce stress changes in the reservoir and caprock, potentially leading to fracturing and fault reactivation, which compromise seal integrity and may result in hydrogen leakage. Additionally, changes in pore pressure can cause reservoir compaction, leading to surface subsidence and further stress on the caprock. Comprehensive geomechanical assessments and monitoring are necessary to ensure long-term storage integrity.

### 8.5. Cushion Gas Requirements and Operational Costs

Establishing a cushion gas is essential to maintain reservoir pressure and facilitate hydrogen withdrawal. In DSAs, up to 80% of the total gas volume may be required as cushion gas, representing a significant capital investment and reducing the proportion of working gas. The interaction between hydrogen and cushion gases can lead to mixing, necessitating additional purification steps during gas retrieval. Optimizing cushion gas composition and volume is vital for economic and operational efficiency.

### 8.6. Site Characterization and Monitoring

Effective hydrogen storage in DSAs demands thorough site characterization, including detailed understanding of porosity, permeability, mineralogy, and caprock integrity. Establishing baseline geochemical and microbial profiles aids in detecting changes due to hydrogen injection. Implementing robust monitoring for pressure, temperature, gas composition, and potential leakage pathways ensures early detection of issues and informs mitigation strategies. Advanced modeling and real-time monitoring technologies are critical components of a successful UHS operation.

Addressing these challenges through interdisciplinary research, advanced modeling, and field-scale pilot projects will be pivotal in realizing the full potential of deep saline aquifers for sustainable hydrogen storage.

### 8.7. Comparative Analysis and Synthesis of Challenges

Table 8 provides a comparative view of the major challenges associated with hydrogen storage in deep saline aquifers, capturing their distinct drivers, consequences, and overlapping interactions. While each domain, chemical, biological, physical, mechanical, or operational poses unique limitations, the key insight from this comparative framework is the extent to which these challenges are interdependent.

The chemical and microbial consumption of hydrogen, for instance, not only results in direct gas loss but also modify the reservoir conditions in ways that affect injectivity and storage dynamics. Similarly, the unfavorable flow characteristics of hydrogen contribute to uneven plume distribution, amplifying exposure to reactive mineral surfaces and microbial niches. Geomechanical risks, while often considered independently, are also shaped by these chemical and flow-related changes, particularly where dissolution or pressure fluctuations alter formation integrity.

Operational constraints, such as cushion gas demand, may seem external to subsurface reactions, yet they influence in situ gas composition and cycling behavior both of which can accelerate or buffer subsurface processes. In turn, all these effects reinforce the need for comprehensive site characterization and ongoing monitoring, not only as diagnostic tools but as predictive instruments to inform adaptive management.

Thus, the comparative matrix emphasizes that addressing any one challenge in isolation is insufficient. Mitigation strategies must be systemic, with integrated modeling and design that accounts for the overlapping physical, chemical, biological, and operational feedback unique to hydrogen storage systems in saline formations.

## 9. Ongoing Initiatives and Future Perspectives for Hydrogen Storage in Deep Saline Aquifers

Research and pilot activities in saline aquifer hydrogen storage are advancing, reflecting both the capacity these formations offer and the technical complexities inherent in converting them into reliable energy reservoirs. Among the pioneering real-world efforts is the Fluxys-led initiative in the Loenhout aquifer (Belgium), where established natural gas storage infrastructure is being adapted for hydrogen injection (Table 9). This karstic limestone reservoir, operational since the 1980s, provides a valuable site for cyclic injection and withdrawal under typical reservoir pressures of 1000–1500 m depth [102].

Researchers from Ghent University have conducted X-ray computed tomography (CT) experiments on Loenhout carbonate cores, demonstrating that hydrogen relative permeability characteristics mirror those of methane, unlike the nitrogen analog, highlighting the need for precise fluid-property treatments in simulation and design [15,103].

Parallel laboratory and pilot-scale investigations are beginning to demonstrate key mechanisms governing hydrogen storage efficiency. Detailed pore-scale X-ray CT studies reveal that hydrogen injection into brine-saturated sandstone cores exhibits mixed capillary and viscous fingering, confirming that heterogeneity could substantially impede sweep efficiency [15]. Wettability and hysteresis effects have emerged as critical factors: numerical modeling informed by relative permeability hysteresis shows that recovery factors decline by ~45–78% depending on wettability state, underscoring the importance of water-wet conditions for preserving hydrogen in-place [38].

Biogeochemical and geomechanical issues also demand careful evaluation. Experiments combining natural gas with 2% hydrogen over multi-month periods indicate microbial activity could generate secondary gases (e.g., methane, hydrogen sulfide), potentially altering gas composition and raising safety concerns. Modeling efforts, including the DROGEOZeit initiative in Germany are investigating caprock response, multiphase flow, and cycling integrity under operational uncertainty [104,105]. Compositional simulations of hydrogen/CO_2_ cushion mixtures show cushion-gas selection plays a major role in balancing buoyancy and maintaining target purity.

Despite growing technical understanding, only a few field-scale hydro-equivalent storage pilots are presently in saline aquifers (Table 8). In contrast, salt-cavern projects such as France’s HyPSTER (44 *t* H_2_ pilot) and the larger-scale U.S. ACES Delta site (300 GWh hydrogen equivalent) have advanced farther toward commercialization [105]. However, saline aquifers offer significantly greater storage volumes with higher cushion-gas volumes, operational risks, and complex heterogeneity challenges.

In conclusion, deep saline aquifers present immense long-term promises as hydrogen storage reservoirs. Yet, realizing their potential relies on resolving critical technical uncertainties from multiphase flow and geomechanics to microbiology and economics and carrying out strategic pilot demonstrations, both to validate underlying physical models and to de-risk multi-billion-dollar energy infrastructure investments.

## 10. Conclusions

Deep saline aquifers represent a promising yet technically challenging medium for large-scale underground hydrogen storage. This study highlights the intricate interplay of physical, chemical, biological, geomechanical, and operational factors that govern hydrogen behavior post-injection. Multiphase flow phenomena, including viscous and capillary fingering, coupled with capillary trapping and relative permeability hysteresis, significantly influence hydrogen distribution and recoverability. Geochemical and microbial processes contribute to hydrogen consumption and gas contamination, while cushion gas requirements and operational strategies directly affect storage economics and efficiency. Geomechanical stability, caprock integrity, and induced seismicity risks further underscore the need for rigorous site characterization and monitoring.

Integrated experimental, numerical, and field-scale investigations are essential to address these interdependent challenges. Economic analyses reveal that hydrogen recovery efficiency and cushion gas optimization are critical levers for reducing levelized costs of storage. Pilot studies, such as those conducted in Loenhout and other European and U.S. sites, provide valuable insights into operational strategies, gas–brine–rock interactions, and monitoring protocols.

In conclusion, realizing the full potential of deep saline aquifers for hydrogen storage requires a holistic, coupled-process approach that combines reservoir characterization, geochemical and microbial assessments, geomechanical modeling, and techno-economic optimization. By bridging knowledge gaps and deploying strategic pilot projects, DSAs can become a viable and scalable solution for supporting the global transition to a decarbonized energy system.

Even with recent advancements, there are still several important unknowns that need careful research before commercial hydrogen storage in saline aquifers can be implemented. The goal of future research should be to identify the mechanisms underlying microbial inhibition, such as the creation of specific biocides, pH management techniques, and microbial community engineering methods to inhibit sulfate reduction and methanogenesis without endangering the integrity of reservoirs. Establishing pressure management techniques that guarantee caprock stability and reduce induced seismicity during cyclic injection and withdrawal operations requires parallel efforts. Additionally, to ensure containment, identify early leakage, and improve predictive models at the field scale, integrated monitoring and validation frameworks that combine geophysical surveys, geochemical tracers, and data-driven analytics will be crucial.

In order to lower parameter uncertainty and increase the scalability of subsurface performance forecasts, coupling reactive transport models with machine learning and real-time sensor data is another top priority. In order to create predictive tools that accurately depict the complexity of hydrogen behavior in natural formations, interdisciplinary cooperation between geoscientists, microbiologists, and systems engineers will be essential. Lastly, the rate at which underground hydrogen storage can move from pilot projects to operational infrastructure within the larger energy transition will depend on techno-economic optimization—through better cushion gas design, hybrid storage strategies, and supportive policy frameworks.

Deep saline aquifers can eventually be used as a secure, effective, and financially feasible basis for massive hydrogen storage and decarbonized energy systems by tackling these scientific and practical issues.

## Figures and Tables

**Table 1:** Comparative attributes of hydrogen storage methods. (Adapted from: [13,19,20,21,22,23,24]).

Storage Method	Typical Scale	Volumetric Energy Density (MJ/L)	Purity Upon Retrieval	Capital Cost	Cycle Frequency	Response Time	Storage Duration	Commercial Maturity
Compressed gas (700 bar)	Small-medium	3–5	Very high	Moderate to high	High	Seconds to minutes	Hours to days	High
Liquid hydrogen	Medium	8.5	Very high	High	Medium	Minutes	Days to weeks	Medium
Solid-state (e.g., hydrides)	Small	6–10 (effective)	High	High	Low	Minutes to hours	Days to weeks	Low
Salt caverns	Large	~6 (gas-phase)	High	Very high	High	Minutes	Weeks to months	High
Depleted oil/gas reservoirs	Very large	~4–6 (gas-phase)	Moderate	Moderate	Low-medium	Hours	Months to years	Medium
Saline aquifers	Very large	~4–5 (gas-phase)	Moderate to low	Moderate	Low	Hours to days	Months to years	Low
Abandoned mines	Medium	Variable	Low to moderate	Low to moderate	Low	Hours to days	Variable	Very low

**Table 2:** Key parameters and processes in dynamic modeling of hydrogen storage in saline aquifers.

Category	Key Parameters/Processes	Modeling Role	Typical Data Sources
Reservoir architecture	Porosity, permeability, anisotropy, heterogeneity	Govern fluid flow, gas trapping, and sweep efficiency	Well logs, core analysis, seismic data
Fluid properties	Hydrogen density, viscosity, compressibility	Affects injection pressure, buoyancy, and plume mobility	PVT experiments, EOS modeling
Multiphase flow	Relative permeability, capillary pressure, hysteresis	Controls phase interactions, residual trapping, and saturation fronts	Core flooding, lab drainage/imbibition tests
Operational cycling	Injection/withdrawal timing, flow rates	Determines pressure evolution and recovery over time	Scenario simulations, historical production data
Cushion gas dynamics	CH_4_/N_2_/CO_2_ interactions, mixing, miscibility	Influences recovery, mixing losses, and injectivity	Thermodynamic modeling, compositional simulations
Geochemical interactions	Microbial activity, hydrogen solubility, mineral reactions	Impacts include hydrogen loss, porosity changes, and long-term stability	Geochemical assays, reactive transport models
Uncertainty analysis	Parameter sensitivity, stochastic realization	Supports risk assessment and optimization of storage strategy	Monte Carlo simulations, ensemble modeling

**Table 3:** Key practical factors influencing hydrogen storage capacity in deep saline aquifers.

Factor	Description	Impact on Capacity	References
Geological heterogeneity	Variability in porosity, permeability, and seal integrity across the reservoir	Causes uneven hydrogen distribution and trapping	[4,19,73]
Cushion gas requirements	Non-recoverable gas volume needed to maintain pressure and enable withdrawal	Occupies 50–75% of pore volume	[74,75,76,77]
Wettability and hysteresis	Capillary effects and flow hysteresis during cyclic injection–production cycles	Reduces recovery efficiency and residual trapping	[78,79]
Operational constraints	Wellbore integrity, injectivity, surface compression capacity	Limits storage rate and scale	[73,80,81]
Economic feasibility	Costs of cushion gas, material compatibility, and long-term monitoring	Influences project viability and scalability	[41,82]

**Table 4:** Comparative overview of factors affecting hydrogen injection and plume evolution in deep saline aquifers.

Factor	Origin	Primary Impact	Implications for Storage
Multiphase flow and fingering	Fluid dynamics	Unstable front, poor sweep efficiency	Requires advanced multiphase modeling and heterogeneous mapping
Capillary trapping and hysteresis	Capillarity, saturation cycles	Residual hydrogen, reduced recovery	Demands hysteresis modeling in simulation
Geochemical interactions	Rock–fluid chemistry	Mineral alteration, porosity/permeability shifts	Must include reactive transport models
Microbial activity	Biogeochemical processes	Hydrogen loss, gas quality degradation, infrastructure corrosion	Calls for microbial monitoring and mitigation strategies
Cushion gas mixing	Operational strategy	Hydrogen dilution, purity reduction	Trade-off between injectivity and withdrawal quality
Induced seismicity	Geomechanical stress	Reservoir destabilization, leakage risk	Necessitates geomechanical simulation and risk management
Boundary conditions	Geological and operational	Plume migration, breakthrough behavior	Essential for accurate modeling and site design
Driving Forces and fluid properties	Physical properties of fluids	Buoyancy and mobility-driven instability	Controls injection strategy and plume predictability

**Table 5:** Comparison of the main conclusions from recent research.

Study	Focus Area	Reported H_2_ Recovery (%)	Microbial H_2_ Loss (%)	LCOS (USD kg^−1^ H_2_)	Key Insights
Heinemann [19,50]	Geological and physical mechanisms	60–80	-	-	Permeability and trap geometry were found to be the main factors
[82]	Techno-economic overview	55–75	-	2.3–3.4	highlighted reservoir uncertainty and the cost of cushion gas as major cost drivers.
[8]	Biogeochemical modeling	50–70	20–45	-	demonstrated a close relationship between hydrogen loss and microbial kinetics.
[38,93]	Cushion gas optimization	70–85	-	1.8–2.6	demonstrated how methane and nitrogen cushions enhance recuperation and lower LCOS.
[11,96]	Economic feasibility (U.S. case study)	-	-	1.3–2.2	Because saline aquifers require cushion gas, they are more expensive than salt caverns.
This Study	Integrated hydro–biogeochemical–economic synthesis	45–85	10–50	1.3–3.4	offers a cohesive evaluation that connects economic, microbial, and flow aspects for scalability.

**Table 6:** Sensitivity of key economic parameters on LCOS for saline aquifer hydrogen storage.

Parameter	Range Evaluated	Impact on LCOS (±%)
Cushion gas cost	±25%	±32%
Hydrogen recovery efficiency	±15%	±19%
Capital cost escalation	±20%	±15%
Discount rate	5–10%	±11%
O&M costs	±20%	±8%

**Table 7:** Roadmap for developing hydrogen storage in deep saline aquifers (Adapted from [99,100]).

Timeframe	Strategic Focus	Key Actions
2025–2030	Site selection and pilot testing	Geologic screening; H_2_–brine–rock interaction studies; small-scale field pilots; monitoring setup
2030–2040	Economic scaling and regulatory alignment	Cushion gas substitution; LCOS optimization; harmonized permitting; performance standards
2040–2050	Full-scale deployment and public engagement	Commercial hubs; community outreach; real-time data sharing; integration into H_2_ infrastructure

**Table 8:** Summary of key challenges in hydrogen storage in deep saline aquifers.

Challenge	Primary Concern	Main Effects	Cross-Domain Interactions
Geochemical reactions	Hydrogen consumption via redox reactions with minerals	H_2_ loss, formation of H_2_S, porosity alteration	Affects geomechanics (porosity/permeability), interacts with microbial activity
Microbial activity	Bioconversion of hydrogen to CH_4_ or H_2_S by subsurface microbes	H_2_ loss, biofouling, gas contamination	Enhanced by brine chemistry; affects injectivity and reservoir quality
Fluid dynamics	Low viscosity of hydrogen causes poor sweep efficiency and residual trapping	Reduced retrievable gas volume, early breakthrough	Influences reactive surface area (geochemistry), creates zones for microbial colonization
Geomechanical stability	Pressure cycling may induce fault activation or caprock damage	Potential leakage, reduced sealing capacity	Influenced by mineral dissolution/precipitation; affects monitoring design
Cushion gas requirements	High cushion gas volume needed to stabilize pressure and enable withdrawal	Lower working gas capacity, cost increase, gas mixing	Cushion gas may interfere with microbial or chemical interactions; affects economic feasibility
Site characterization	Need for detailed geological, geochemical, and biological understanding	Baseline data for monitoring and modeling	Enables risk assessment in all other domains; essential for integrated modeling

**Table 9:** Selected international projects and initiatives targeting hydrogen storage in saline aquifers and salt caverns.

Project	Country	Geologic Type	Status	Notes
Loenhout aquifer	Belgium	Karstic carbonate	Pilot-ready	Adapting existing cyclic natural gas storage; permeability experiments show H_2_ vs. CH_4_ flow similarities [102,103]
GEOZeit	Germany	Saline aquifer	In development	Advanced modeling of integrity under cycling and uncertainty
HyPSTER	France	Salt cavern	Underway pilot	1 MW electrolyzer, 44 *t* hydrogen injected for seasonal storage
ACES delta	USA	Salt cavern	Construction stage	Multi-GWh hydrogen capacity; benchmark for cavern-scale storage

## Data Availability

No new data were created or analyzed in this study. Data sharing is not applicable to this article.
